# Cultivation of Sponge-Associated Bacteria from *Agelas sventres* and *Xestospongia muta* Collected from Different Depths

**DOI:** 10.3390/md17100578

**Published:** 2019-10-11

**Authors:** Anak Agung Gede Indraningrat, Sebastian Micheller, Mandy Runderkamp, Ina Sauerland, Leontine E. Becking, Hauke Smidt, Detmer Sipkema

**Affiliations:** 1 Laboratory of Microbiology, Wageningen University & Research, Stippeneng 4, 6708 WE Wageningen, The Netherlands; s.micheller@online.de (S.M.); mandy.runderkamp@wur.nl (M.R.); ina.sauerland@wur.nl (I.S.); hauke.smidt@wur.nl (H.S.); 2 Faculty of Medicine and Health Science, Warmadewa University, Jln Terompong 24, Denpasar 80239, Bali, Indonesia; 3 Marine Animal Ecology Group, Wageningen University & Research, Droevendaalsesteeg 1, 6708 PB Wageningen, The Netherlands; lisa.becking@wur.nl; 4 Wageningen Marine Research, Wageningen University & Research, Ankerpark 27, 1781 AG Den Helder, The Netherlands

**Keywords:** sponges, cultivation, bacteria, depth

## Abstract

Sponge-associated bacteria have been mostly cultured from shallow water (≤30 m) sponges, whereas only few studies targeted specimens from below 30 m. This study assessed the cultivability of bacteria from two marine sponges *Xestospongia muta* and *Agelas sventres* collected from shallow (<30 m), upper mesophotic (30–60 m), and lower mesophotic (60–90 m) reefs. Sponge-associated bacteria were cultivated on six different media, and replicate plates were used to pick individual colonies or to recover the entire biomass. Prokaryotic community analysis was conducted using Illumina MiSeq sequencing of 16S rRNA gene amplicons. A total of 144 bacterial isolates were picked following a colony morphology coding scheme and subsequently identified by 16S rRNA gene sequence analysis. Sponge individuals at each depth-range harboured specific cultivable bacteria that were not retrieved from specimens collected at other depths. However, there were substantial differences in the number of colonies obtained for replicate sponges of the same species. In addition, source of inoculum and cultivation medium had more impact on the cultured prokaryotic community than sample collection depth. This suggests that the “plate count anomaly” is larger than differences in sponge-associated prokaryotic community composition related to depth.

## 1. Introduction

Sponges are among the prominent producers of secondary metabolites in marine environments with approximately 5000 compounds described, which contribute to approximately 30% of the known marine natural products to date [1]. Many of these sponges’ natural products show promising therapeutic applications, such as antimicrobial, anticancer, antitumor, and anti-inflammatory activity [2,3]. In addition, it has turned out that many of the bioactive compounds found in sponges are of microbial origin, indicating that microbial communities associated with sponges may play a key role for biosynthesizing these bioactive molecules [4,5,6].

Bacteria constitute a major fraction of the prokaryotic community of most marine sponges, and many of these sponge-associated bacteria are of ecological and biotechnological importance [7,8]. Sponge-associated bacteria have been attributed to play an important role in various nutrient cycles including those related to carbon [7], nitrogen [9,10], sulphur [11,12] and phosphorus metabolism [13]. In addition, sponge-associated bacteria are involved in host-defence by producing biologically active compounds to protect their host from predation, fouling organisms and microbial infections [7,8]. Yet, the true potential of these bioactive compounds produced by sponge-associated bacteria is largely unexplored since the majority of sponge-associated bacteria has remained recalcitrant to cultivation in the laboratory [14].

Despite the fact that currently only a small percentage of sponge-associated bacteria is accessible through culturing, cultivation remains an important approach to assess a bacterial strain’s biotechnological potential [14,15]. To date, the cultivable sponge-associated bacteria are mainly members of the phyla Actinobacteria, Bacteroidetes, Firmicutes, Proteobacteria (Alpha- and Gamma-, and to a lesser extent also Deltaproteobacteria), Planctomycetes and Verrucomicrobia [14,16,17,18,19,20]. Of these phyla, members of the Actinobacteria are recognized as prominent producers of antimicrobial substances [21]. In addition, antimicrobial activities also are frequently detected from cultivable Proteobacteria and Firmicutes from sponges, represented mainly by the genera Pseudovibrio and Bacillus [22].

Sponges are widely distributed over a large bathymetric range from shallow water (<30 m) to the upper mesophotic (30–60 m) and lower mesophotic (60–200 m) zone and the deep-sea [23,24,25,26]. To date, however, the majority of studies on the marine sponge prokaryotic community composition has been done with samples collected at shallow depth (<30 m) [27,28], while data generated from sponges from a greater depth (>30 m) is sparse [23,24,25]. This discrepancy is mainly caused by technical constraints to obtain sponge samples from greater depth, as specialized equipment such as technical diving gear and submersibles are required, which in most cases are expensive or even inaccessible [29,30,31]. The few studies that have reported on the impact of depth on the sponge prokaryotic community composition using culture-independent studies showed that the composition changes when depth increases, which is mainly explained by environmental factors related to depth, such as light intensity and nutrient availability [23,24]. Additionally, unique microbial gene clusters encoding for the biosynthesis of secondary metabolites have been detected from deep-sea sponges [32]. This implies that the deeper living sponges and their associated bacteria harbour an additional biotechnological potential beyond their shallow counterparts [33].

*Xestospongia muta* and *Agelas sventres* are conspicuous sponges in the Caribbean Sea and have a considerable depth span, ranging from 2 m to reach down to approximately 100 m [24,34,35]. These two species are characterized by dense and diverse bacterial communities and are classified as “high microbial abundance sponges” [23,24]. Both sponges are also rich in secondary metabolites with a remarkable array of biological activities including antimicrobial activity [36,37]. A previous cultivation study aiming to grow bacteria from *X. muta* from shallow water recovered bacterial isolates assigned to Actinobacteria, Bacteroidetes, Firmicutes and Proteobacteria [38]. Conversely, no bacterial cultivation study has been reported for *A. sventres*.

In this study we investigated the cultivability of sponge-associated bacteria of *X. muta* and *A. sventres* collected across a depth gradient to assess to what extent sponges from different depths yield different bacterial isolates given the different ecological settings at different depths namely shallow water (<30 m depth), upper mesophotic zone (30–60 m) and lower mesophotic (60–90 m). Therefore, we hypothesized that sponges from different depths could harbour different cultivable bacteria, which ultimately may represent biological sources with different biological activities needed for the development of novel pharmaceuticals. We applied different types of cultivation media to capture as much variation of cultivable bacteria from sponge inocula as possible. Furthermore, to be able to perform high-throughput identification of the isolates, we collected total colony material for each growth medium used and performed 16S ribosomal RNA (rRNA) gene amplicon sequencing of the total cultured biomass.

## 2. Results

Six different agar-based growth media cumulatively yielded 650 and 3024 bacterial colonies from nine *X. muta* samples of three depth categories (deep, middle, shallow) and six *A. sventres* samples of two depth categories (middle and shallow), respectively (Table 1). Relatively similar numbers of colonies were observed from each depth range for *X. muta* and also for *A. sventres* (Table 1 and Table S3). However, from *A. sventres* individuals, more colonies were observed than from *X. muta* individuals. Furthermore, substantial intraspecific variation in the number of colonies was observed for individuals from the same depth range, both for *X. muta* and *A. sventres* (Table 1).

**Figure 1:**
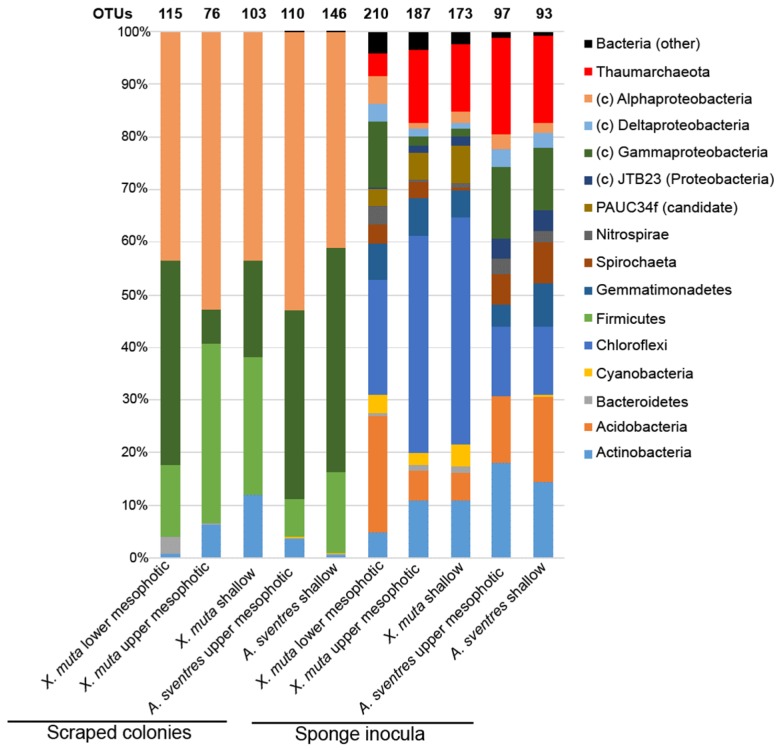
Distribution of prokaryotic phyla based on total reads obtained from Illumina MiSeq sequencing of 16S rRNA genes amplified from scraped colonies and sponge inocula. Reads derived from biomass recovered from plates and sponge inocula were pooled per depth category. The total numbers of operational taxonomic units (OTUs) per pool are indicated on top of each bar. Phyla with an average relative abundance lower than 0.5% in all samples (Poribacteria, SBR1093, Tectomicrobia, Nitrospinae, Deinococcus Thermus, Verrucomicrobia, Betaproteobacteria (class level) and bacterial reads not assigned to a phylum) were collapsed under “bacteria (other)”. The phylum Proteobacteria is displayed at the class level (Alpha-, Gamma-, Delta- and JTB23). PAUC34f is a candidate phylum.

Illumina MiSeq sequencing of sponge sample inocula and the bacterial colonies recovered from agar plates yielded a total of 5,545,747 high-quality reads, which clustered into 791 operational taxonomic units (OTUs, defined as unique amplicon sequence variants at a detection threshold of 0.1% relative abundance per sample, and allowing for one mismatch with sequences occurring at lower abundance), with 371 OTUs being identified from scraped plates (Table S1). The biomass recovered from plates inoculated with samples of *X. muta* consisted mainly of representatives of four phyla, namely Proteobacteria (Alpha- and Gamma-), Firmicutes, Actinobacteria and Bacteroidetes (Figure 1). A high relative abundance (>40%) of the class Alphaproteobacteria was observed for all bacterial biomass recovered from plates inoculated with samples of *X. muta* from all depths, whereas isolates belonging to the remaining phyla/classes varied more with depth. Firmicutes was the second most abundant phylum with 13.6 ± 1.3 % of the reads in the biomass recovered from plates inoculated with lower mesophotic *X. muta* samples, 34.1 ± 3.1% in upper mesophotic and 26.0 ± 1.5% in shallow samples. The class Gammaproteobacteria represented 39.0 ± 1.1% from the reads of the lower mesophotic samples and lower relative abundances of 6.5 ± 0.2% and 18.5 ± 0.7% in the upper mesophotic and shallow samples, respectively. Furthermore, the percentage of Actinobacteria in the cultured fraction was 12.0 ± 1.1% in shallow samples and decreased to 6.4 ± 0.7% and 0.66 ± 0.07% as depth increased.

**Figure 2:**
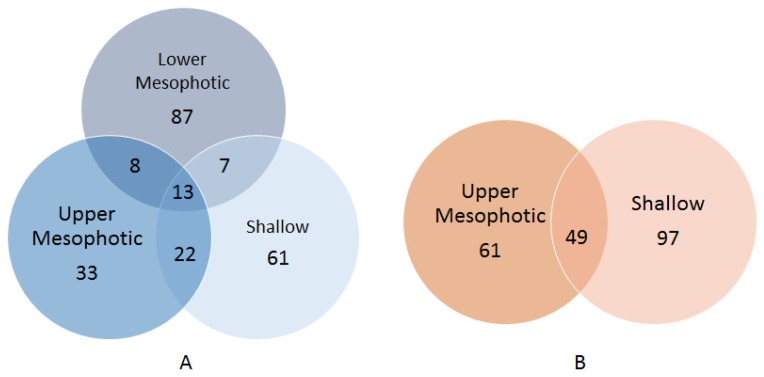
Venn diagrams with the numbers of OTUs retrieved from colonies scraped off agar media shared between *X. muta* (**A**) and *A. sventres* (**B**) from different depth categories.

Among biomass recovered from plates inoculated with samples of *X. muta*, 13 OTUs were obtained from all depth categories (Figure 2A), which were predominantly affiliated with the genus Pseudovibrio (Table S2). OTUs classified as Mycobacterium (Actinobacteria), Salegentibacter, Tenacibaculum (Bacteroidetes), Fictibacillus, Marinococcus, Planococcaceae (Firmicutes), Mameliella, Roseomonas (Alphaproteobacteria), Alteromonas and Alcanivorax (Gammaproteobacteria) were present in the biomass recovered from plates inoculated with samples of lower mesophotic *X. muta*, but were absent from their upper mesophotic and shallow *X. muta* counterparts. Furthermore, OTUs assigned to Caulobacteraceae, Brevundimonas and Paracoccus (Alphaproteobacteria) were only present in biomass recovered from plates inoculated with upper mesophotic *X. muta* samples. Lastly, OTUs assigned to Brachybacterium, Arthrobacter, Rothia, Kocuria (Actinobacteria), Labrenzia, Altererythrobacter (Alphaproteobacteria), Parahaliea, Exiguobacterium, and Lactobacillus (Firmicutes) were only found in the bacterial biomass recovered from plates inoculated with shallow samples. The only OTU overlapping between lower mesophotic *X. muta* inocula and the corresponding bacterial biomass on agar plates was OTU194 (Halomonas) (Table S2). However, this single overlapping OTU was represented only by 131 reads in one inoculum (XM14) and accounted for 0.13% of the total reads in that inoculum.

The bacterial biomass recovered from plates inoculated with samples of of *A. sventres* collected from shallow and the upper mesophotic zone comprised three main phyla: Proteobacteria (Alpha- and Gamma-), Firmicutes and Actinobacteria, which cumulatively contributed to 99.5% of the total prokaryotic community. The relative abundance of the class Alphaproteobacteria and the phylum Actinobacteria increased from 41.0 ± 1.7% and 0.45 ± 0.04% in shallow specimens, respectively, to 53.0 ± 1.9% and 3.6 ± 0.2%, in the upper mesophotic specimens. Conversely, the percentages of Gammaproteobacteria and Firmicutes decreased from 42.6 ± 1.2% and 15.4 ± 1.7% in shallow specimens to 35.8 ± 1.7% and 7.3 ± 0.7%, respectively, in the upper mesophotic specimens. The remaining 0.5 ± 0.01% of the bacterial reads in biomass recovered from plates inoculated with samples of both shallow and upper mesophotic *A. sventres* specimen belonged to Bacteroidetes, Betaproteobacteria, Cyanobacteria and Verrucomicrobia.

Forty-nine OTUs were shared between biomass recovered from plates inoculated with samples of upper mesophotic and shallow specimens of *A. sventres* (Figure 2B). These were assigned to the genera Janibacter (Actinobacteria), Synechococcus (Cyanobacteria), Labrenzia, Pseudovibrio, Ruegeria (Alphaproteobacteria), Limnobacter (Betaproteobacteria), Microbulbifer and Endozoicomonas (Gammaproteobacteria). OTUs assigned to Corynebacteriales, Nocardia, Rhodococcus (Actinobacteria), Muricauda, Flavobacteriaceae (Bacteroidetes), Paracoccus, Sphingomonas (Alphaproteobacteria) and Alcanivorax (Gammaproteobacteria) were present in bacterial biomass recovered from plates inoculated with samples from the upper mesophotic *A. sventres* specimens, but absent from their shallow counterparts, whereas OTUs assigned to Brucellaceae, Erythrobacter, Mesorhizobium, Rhizobium, Phyllobacterium (Alphaproteobacteria), Enterobacteriaceae, Vibrionaceae, Vibrio, Escherichia-Shigella (Gammaproteobacteria) were only found in biomass recovered from plates inoculated with shallow *A. sventres* samples (Table S2). Limited overlap was found between OTUs in the inocula and biomass recovered from plates inoculated with samples of *A. sventres*. OTU1404 (Pseudovibrio), and OTU514 and OTU620 (Endozoicomonas) were the three OTUs that overlapped between inocula and biomass recovered from plates inoculated with samples from the upper mesophotic zone. OTU1404 was only present in inoculum AS2 and accounted for 0.2% of the reads in that inoculum. OTU514 and OTU620 were found in two inoculums (AS2 and AS3) and on average accounted for 1% and 0.1% of the reads from the two inoculums respectively. OTU514 and OTU816 (Synechococcus) were the two OTUs shared between inocula and biomass recovered from plates inoculated shallow samples. OTU514 was detected in two inoculums (AS6 and AS10) and on average contributed to 1% of the total reads in these two inoculums. While, OTU816 was only present AS6 and accounted for 0.3% of the reads in that inoculum. 

**Figure 3:**
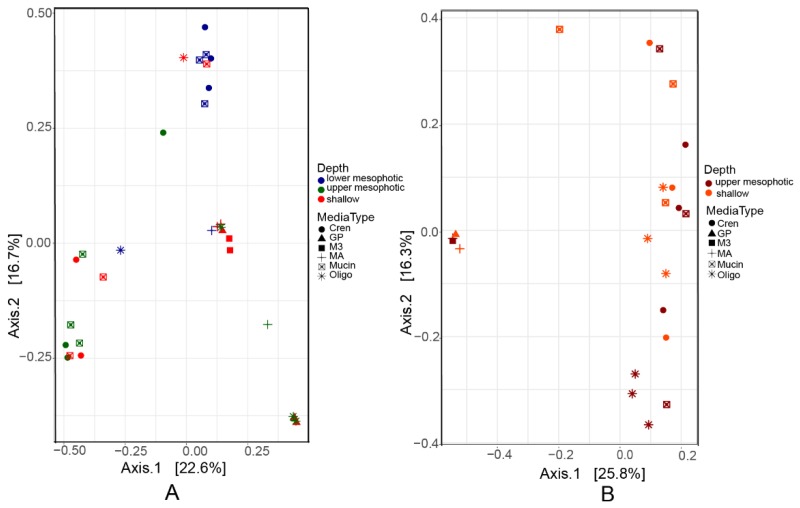
Principal coordinate analysis (PCoA) plots based on Bray–Curtis distance of bacterial composition colored based on depth range from bacterial biomass recovered from plates inoculated with samples of (**A**) *X. muta* and (**B**) *A. sventres*. Media type indicates the agar media on which these baterial colonies were grown (OLIGO, Mucin, Crenarchaeota, M3, GP, MA1/10).

Multivariate analysis using PCoA based on pairwise Bray–Curtis distances showed differences in prokaryotic composition of the biomass recovered from plates inoculated with different samples (Figure 3). The sponge (source of inocula) contributed to 10% (permutation ANOVA (PERMANOVA), *p* = 0.001) of the difference in the scraped biomass (Table 2). In addition, depth explained 10% and 6% of the differences in the biomass recovered from plates inoculated with samples of in *X. muta* and *A. sventres*, respectively. The growth medium used significantly affected the bacterial colonies recovered by cultivation (*p* = 0.001) and explained 31% and 60% of the variation observed of the biomass recovered from plates inoculated with samples of *X. muta* and *A. sventres*, respectively. This is partly explained by the uneven distribution of the numbers of colonies obtained on different growth media. For example GP, M3 and 1/10 MA cumulatively yielded only 1% of the total colonies found in *A. sventres*, and 10% of total bacterial colonies of *X. muta*. From both sponges, the highest numbers of colonies were obtained from CR, OLIGO and Mucin agar media (Table 1 and Table S3).

**Figure 4:**
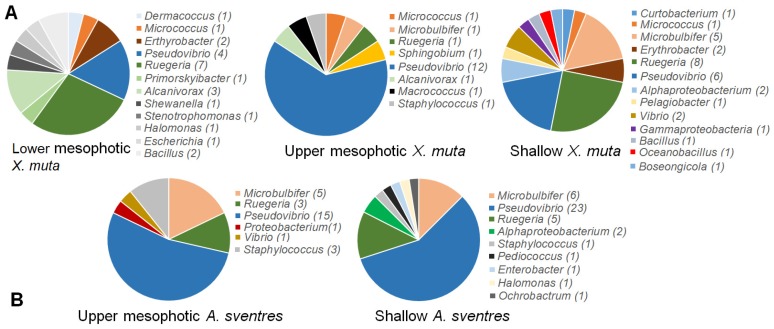
Distribution of picked bacterial colonies from agar plates at the genus level based on 16S rRNA gene sequences. The numbers in parentheses following genus names indicate the number of isolates. Panel (**A**) shows the distribution of colonies in *X. muta* and panel (**B**) represents colonies from *A. sventres*.

**Figure 5:**
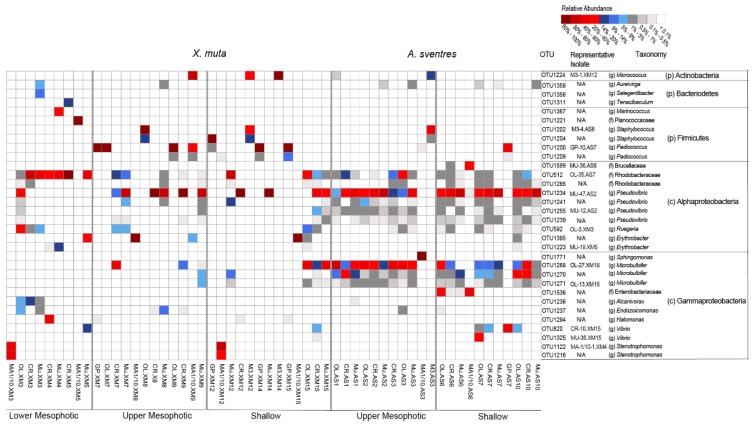
Heatmap representing the most abundant OTUs (average relative abundance ≥ 0.25%) across all samples derived from biomass recovered from plates inoculated with samples of *X. muta* and *A. sventres*. Samples were labelled based on their cultivation media (i.e., OL = OLIGO, CR = Crenarchaeota, Mu = Mucin and MA1/10 = 1/10 diluted Marine Agar, GP = Gram Positive, M3) followed by their sponge specimen code and subsequently were grouped according to their depth category (depth, middle, shallow). For each OTU, a representative isolate from picked colonies is indicated if recovered by picking colonies (otherwise N/A = Not Available, if representative isolates were not found). The letter in parentheses indicates the best taxonomy assignment available for each OTU: g (genus), f (family), c (class), p (phylum).

The replicate plates dedicated for picking colonies yielded a comparable total number of colonies as the plates for recovering the total biomass growing on plates (Table 1 and Table S3). Overall, 76 bacterial colonies from *X. muta* and 68 bacterial colonies of *A. sventres* were picked from agar media and survived after re-streaking on agar media and cultivation in corresponding liquid media. These 144 colonies represented 40 and 16 colony morphology codes (CMCs), respectively (Figure S1 and Table S4). In *X. muta*, Pseudovibrio (22 isolates) and Ruegeria (16) were the two most common genera picked from all agar plates (Figure 4). Likewise in *A. sventres*, the three most frequently picked genera were Pseudovibrio (38), Microbulbifer (7) and Ruegeria (7). Furthermore, sequence comparison of these picked isolates with Illumina MiSeq sequences showed matches only with OTUs from the scraped colonies, but not with OTUs found in sponge inocula (data not shown). Among the most frequently picked colonies (Figure 5), a number of bacterial taxa were consistently detected irrespective of the media used for cultivation, including Rhodobacteraceae (OTU512 and OTU1265), Pseudovibrio (OTU1234 and OTU1255), and Ruegeria (OTU592).

## 3. Discussion

This study investigated the impact of depth on the cultivability of bacteria associated with the sponges *X. muta* and *A. sventres*. Irrespective of their depth category, Proteobacteria (Alpha- and Gamma-), Actinobacteria and Firmicutes were bacterial phyla dominantly detected from the cultivable fraction of both sponges. This observation is in contrast to a cultivation-independent assessment of the prokaryotic communities of these sponges where depth was found to have a significant impact on the associated communities [39]. However, this discrepancy can be explained by the lack of similarity between the isolates obtained and the bacteria present in the original sponge inocula. The latter is a recurring issue in attempts to isolate dominant representatives of sponge-associated bacteria that reside in the inner part of the sponge tissues (mesohyl) [14,38,40,41,42] and has often been explained by the recovery of isolates that were present in low numbers in the canal systems and choanocyte chambers of sponges before processing [14,16,40]. The failure to recover the bacteria abundant in the sponge in the laboratory stresses that new cultivation approaches where metabolic capacities of sponge-associated bacteria—partly unveiled by recent metagenomics studies—are integrated need to be implemented [43,44,45,46,47].

The original depth of the sample significantly affected the bacterial community cultured for *X. muta* and some genera were exclusively detected in the biomass recovered from plates inoculated with lower mesophotic specimens of *X. muta*, such as Alcanivorax and Alteromonas. These genera have also previously been isolated from deep-sea sponges and deep marine habitats [48,49] and have been associated with oil and mucus degradation [50]. On the other hand, some OTUs assigned to Altererythrobacter, Arthrobacter, Brachybacterium, Rothia and Kocuria were exclusively detected in the biomass recovered from plates inoculated with shallow *X. muta* specimens. Differences in cultivable bacteria recovered from *X. muta* inoculums between shallow and lower mesophotic depth hint at the influence of ecological conditions that separate specimens from the two depths. The shallow reef habitat is more exposed to light and tends to have lower nutrient contents compared to lower mesophotic reefs [29,51]. These differences could affect composition of bacteria that reside on the sponge’s tissues and the seawater filtered by the sponges and consequently affect the colonies that can be cultured on agar media. Other studies have shown that depth is indeed an important driver for the diversity of cultivable bacteria from sea water collected from the mesophotic zone to the deep sea [52,53]. Conversely to *X. muta*, no significant effect of depth was found for the bacteria recovered by cultivation from *A. sventres*.

Isolates of the genus Pseudovibrio were consistently detected in the scraped bacterial colonies of all samples and all depths. Pseudovibrio spp. can be vertically transmitted via sponge larvae [54] and have been recovered from a large number of different sponges across the globe [55]. Our results also show that along vertical gradients Pseudovibrio spp. are generally recovered from sponges. In addition to Pseudovibrio, members of the genera Ruegeria and Microbulbifer were also among the most abundant isolates from all sponge specimens, and these two genera have also been frequently reported from other cultivation studies of bacteria from marine sponges [56,57]. Ruegeria has been associated to facilitating cell-to-cell communication between bacteria and the sponge host [58,59], but the fact that these bacteria were not detected in the original samples casts some doubt on this hypothesis. The genus Microbulbifer has been associated with the production of paraben (para-hydroxybenzoate) compounds that play a role as chemical mediator of interactions between microbial associates in marine sponges [60].

Although our aim was not to compare the impact of different growth media (but rather to obtain a diversity of isolates), quantitative differences in the numbers of colonies obtained on different media were striking. An aspect that may have affected the cultivable bacteria from the different sponge samples is the viability of the bacteria in the original samples [61]. A recent study showed that sponge individuals respond differently to the addition of cryoprotectant agents (also used in our experimental setup) leading to large intraspecific variation in viability of sponge cells [62]. Sponges are sensitive and transport from the natural habitats to the laboratory may cause cell damages which could possibly lead to low recoverability of bacterial isolates on agar media [61,63]. In addition, variability of bacterial cell densities in the sponges samples may also have affected the total number colonies obtained on agar media [41,61]. Nevertheless, these quantitative differences were not so much reflected in the bacterial species richness obtained from different individuals of the same species. In other words, the number of colonies belonging to the same species increased rather than the actual number of different species (Table S1).

Differences in total number of colonies may partly be explained by differences in chemical composition. Lower numbers of colonies were obtained from media M3, GP and MA1/10; likely because these media contained lower salt concentrations compared to OLIGO, Mucin and Crenarchaeota. Lower salt concentrations may have affected the recovery of isolates from sponges and consequently limited the number of bacteria that can grow on the corresponding agar media. Media M3, GP and MA1/10 were included here to particularly recover specific bacterial colonies associated to Actinobacteria, Gram positive and slow growing bacteria from sponges [40,63,64].

## 4. Materials and Methods

### 4.1. Sample Collection 

*X. muta* and *A. sventres* samples were collected in front of the Substation Curaçao (12°05′04.4″ N 68°53′53.7″ W) from 4–22 November 2015. For the described study, samples were grouped into three categories; shallow (0–30 m), middle (30–60 m) and deep (60–90 m). *X. muta* is characterized by a typical barrel-shape and even surface with individuals found in shallow water often displaying brownish-red colour, while individuals in deep habitats often have pinkish to pale colour. *A. sventres* shows bulbous, massive-lobate to ball form with internal colour of orange and external colour varying from orange to orange-yellow. Figures of each sponge individual collected for this study are available in Supplementary Figure S1. From each of the depth categories, three individual sponge specimens were collected for each of the two species (Table S5). Shallow sponge specimens were collected via SCUBA diving, while middle and deep specimens were taken using a submersible vehicle, the “Curasub”. Upon arrival in the laboratory at Substation Curaçao, any visible debris was removed from the sponge specimens, and they were rinsed three times using artificial seawater (ASW, 33 g/L synthetic sea salt (Instant Ocean Reef Crystals, Aquarium Systems, Sarrebourg, France)) and sponge tissues were cut into pieces of ~0.1 cm^3^ containing both the interior and exterior of the sponge. Pieces of tissue from each specimen were preserved in a 15 mL tube containing 10 mL of RNAlater stabilization solution (Thermo Fisher Scientific, Waltham, MA, USA) and stored at −20 °C. All sponge samples were identified by sequence analysis of the cytochrome oxidase I (COI) gene amplified by PCR from DNA extracted from these pieces (see below for details). The deep *A. sventres* samples were excluded from the dataset since they formed a different clade from the other *A. sventres* samples based on the COI sequence and may represent a different Agelas species. The remaining tissue pieces of each sponge specimen were homogenized with mortar and pestle, and two tissue volumes of sterile artificial seawater were added to obtain a homogeneous cell suspension. The suspensions were aliquoted into sterile cryo-tubes (Corning, New York, NY, USA) by mixing 1 mL of cell suspension with 0.6 mL of 50% sterile glycerol in ASW before storage at −80 °C.

### 4.2. Cultivation Conditions

**Figure 6:**
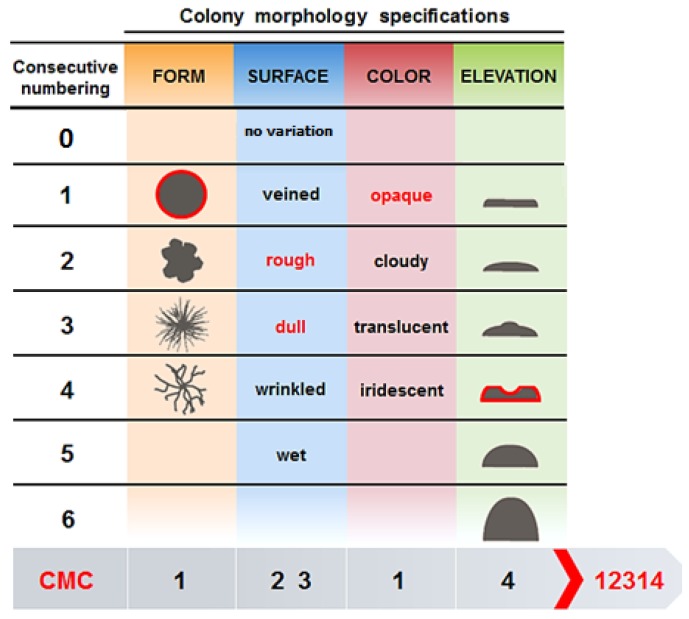
Colony morphology code (CMC) (Adopted from Reference [67], have got the permission from LibreTexts libraries), to categorize the morphology of picked bacterial colonies. For example, a bacterial colony with CMC 12,314 would be described as circular (form), rough and dull (surface), opaque (color) and crateriform (elevation). When a bacterial colony only has one type of surface (without variation), the number 0 is added to the CMC. Therefore, a bacterial colony with CMC 10,512 would be described as circular (form), wet (surface), opaque (color) and raised (elevation).

For each sponge specimen, material from the original glycerol stock was serially diluted to 10^−3^ in ASW. One hundred fifty microliters of dilutions 10^−1^ to 10^−3^ were spread in duplicates onto different agar-based growth media. The following six media were used: (I) marine agar 1/10 (MA1/10) (1 L ddH_2_O, 3.74 g marine broth 2216 (Difco, Detroit, USA), pH 7.6 ± 0.1) [40]; (II) M3 (1 L ddH_2_O, 2 g peptone, 0.1 g asparagine, 4 g sodium propionate, 0.5 g K_2_HPO_4_, 0.1 g MgSO_4_, 0.01 g FeSO4, 5 g glycerol, 20 g NaCl, 0.05 g K_2_Cr_2_O_7_ and 0.015 g nalidixic acid, pH: 7.0 ± 0.1) [64]; (III) OLIGO (1 L ASW, 0.5 g tryptone, 0.1 g sodium glycerol phosphate, 0.05 g yeast extract, pH: 7.6 ± 0.1) [65]; (IV) Gram Positive (GP) (1 L ddH_2_O, 10 g tryptose, 5 g NaCl, 3 g beef extract, 2.5 mL (2-)phenylethanol, pH 7.3 ± 0.1) [63]; (V) Mucin (1 L ASW, 1.0 g Mucin, pH 7.5 ± 0.1) [66]; (VI) Crenarchaeota (1 L ASW, 0.124 g Na_2_CO_3_.2H_2_O, 0.053 g NH_4_Cl, 1 mL tungsten-selenite solution, pH 7.0 ± 0.1) [16]. After autoclaving, media V and VI were supplemented with 1 mL trace metal solution [66], 1 mL phosphate solution [66] and 1 mL vitamin solution (BME vitamins, (diluted 10-fold); Sigma). All media contained 15 g/L of Noble agar (Difco) to produce solid media. Petri dishes were sealed with parafilm and incubated in the dark at 30 °C for 28 days. For each medium, three negative controls were included and inoculated with sterile artificial seawater. The colonies on all plates were counted everyfive days. Replicate plates were subsequently used to either pick individual colonies or to recover the entire biomass. Individual colonies were picked based on their colony morphology code (CMC) (Figure 6) [67]. The CMCs consisted of five-digit numbers that were derived from four main criteria and one sub-criterion: a) form (1= circular, 2 = irregular, 3 = filamentous, 4 = rhizoid), b) surface (0 = no surface variation, 1 = veined, 2 = rough, 3 = dull, 4 = wrinkled, 5 = wet), c) color (1 = opaque, 2 = cloudy, 3 = translucent, 4 = iridescent), d) elevation (1 = flat, 2 = raised, 3 = umbonate, 4 = crateriform, 5 = convex, 6 = pulvinate). A sub-criterion was made for the surface criterion since bacterial colonies may combine different appearances. Therefore, two digit numbers were used to describe the cell surface either based on a single sub-criterion e.g., 02 = rough) or combination of sub-criteria (e.g., 15 = veined and wet).

Colonies (>1 mm in diameter) with different morphologies (CMCs) were isolated from agar plates dedicated for “picking” using sterile toothpicks. A picked colony was labelled based on the medium, the order of picking on the agar plate, and the source of sponge inoculum. Subsequently, selected colonies were transferred to a fresh agar plate and subsequently grown in liquid culture (same media as their original cultivation media without Noble agar). Bacterial isolates were cryopreserved in sterile cryotubes (Corning) by mixing 0.6 mL of growing cultures at an OD600 ≥ 0.2 with 0.4 mL of 50% sterile glycerol in ASW and stored at −80 °C. From a second replicate plate, the total biomass was harvested by adding 1.5 mL of sterile ASW to each agar plate and scraping off the biomass using an L-shaped spreader. Six hundred microliters of the obtained suspension of each dilution from the same inoculum and medium was pooled and equally mixed to be used as the starting material for DNA extraction.

### 4.3. DNA Extraction 

The FastDNA Spin kit for soil (MP biomedicals, Santa Ana, CA, USA) DNA was used to extract total DNA from the sponge inocula as well as from scraped colony material according to the manufacturer’s instructions with the slight modification of conducting 2 times 45 s bead beating (Precellys 24, Montigny-le-Bretonneux, France). Concentration and quality of extracted DNA was checked using a spectrophotometer (DeNovix DS-11, Wilmington, NC, USA), and by electrophoresis on a 1% agarose gel.

### 4.4. Prokaryotic Community Profiling Using 16S rRNA Gene Amplicon Sequencing

The composition of sponge inocula and colony material was assessed by Illumina MiSeq amplicon sequencing of 16S rRNA gene fragments using a two-step amplification procedure [68]. PCR was conducted by using the primer pair 515FY (5′GTGYCAGCMGCCGCGGTAA 3′) [69] and 806RB (5′GGACTACNVGGGTWTCTAAT 3′) [70], where Unitag 1 and Unitag 2 were added to the forward and reverse primer (Table S6), respectively, as previously described [68]. In the first step PCR, 25 μL PCR reactions contained 16.55 μL nuclease free water (Promega, Madison, WI, USA), 5 μL of 5× HF buffer, 0.2 μL of 2 U/μL Phusion hot start II high fidelity polymerase (Thermo Fisher Scientific AG), 0.75 μL of 10 μM stock solutions of each primer, 0.75 μL 10 mM dNTPs (Promega) and 1 μL template DNA (10–20 ng/μL). Amplification was performed at 98 °C for 3 min, followed by 25 cycles at 98 °C for 25 s, 50 °C for 20 s, 72 °C for 20 s and a final extension of 7 min at 72 °C. PCR products were visualized on a 1% (*w*/*v*) agarose gel. Subsequently, 5 μL of these first-step PCR products were used as template in the second PCR reaction to incorporate 8 nt sample specific barcodes as previously described [68]. The second step PCR was performed in triplicate for each sample in 50 μL PCR reactions which contained 31 μL nuclease free water (Promega), 10 μL of 5× HF buffer, 0.5 μL of 2 U/μL Phusion hot start II high fidelity polymerase (Thermo Fisher Scientific AG), 5 μL equimolar mixes of 10 µM forward primer (barcode-linker-Unitag1) and reverse primer (barcode-linker-Unitag2), 1 μL 10mM dNTPs (Promega) and 2.5 µL of the first PCR product. The PCR products were purified using the HighPrep^TM^ PCR clean-up kit (Magbio, London, UK), and quantified using the Quant-iTdsDNA high-sensitivity assay kit (Invitrogen) and the Qubit fluorometer 2.0 (Invitrogen, Grand Island, NY, USA). Samples were pooled in equimolar concentrations to ensure equal representation of each sample [68]. The pooled library was purified, concentrated, and quantified again with the HighPrep^TM^ PCR clean-up kit (Magbio) and the Quant-iTdsDNA high-sensitivity assay kit (Invitrogen). Finally, the library was sequenced at GATC Biotech AG (Konstanz, Germany) by Illumina MiSeq sequencing.

### 4.5. Sequence Data Processing 

Raw DNA sequence data was analyzed using the NG-Tax pipeline (Galaxy version 1.0) [71]. NG-tax was used as previously described [72] with some modifications with respect to the final length of trimmed and concatenated sequences and the version of SILVA database used for taxonomic assignment. Briefly, paired-end libraries were combined, and only read pairs with matching primers and barcodes were retained. Forward and reverse reads were trimmed to 70 nucleotides to avoid overlap in forward and reverse reads. Paired-end trimmed forward and reverse reads were concatenated, and the resulting 140 bp were subsequently used for sequence data processing. Demultiplexing, assignment of OTUs, chimera removal, and taxonomic assignment were performed using default settings of the NG-tax pipeline (Galaxy version 1.0). Reads were ranked per sample by abundance and OTUs (at a 100% identity level) were added to an initial OTU table for that sample, starting from the most abundant sequence until the relative abundance was lower than 0.1%. The final OTU table was created by clustering the reads that were initially discarded as they represented OTUs with relative abundances < 0.1% with the OTUs from the initial OTU table with allowing one nucleotide mismatch (98.5% similarity) [71]. Finally, taxonomic assignment was done by utilizing the SILVA 128 SSU database [73]. OTUs classified as Chloroplasts were removed from the analysis.

### 4.6. Prokaryotic Diversity Analyses

The bar plots of community composition at the phylum level were generated with Microsoft Excel 2016. Other prokaryotic diversity data analyses were performed in R Statistical Software (version 3.4.2) (https://www.r-project.org). Prokaryotic community beta diversity was visualized by principal coordinate analysis (PCoA) based on relative abundance of OTUs after Hellinger transformation and generated using Bray–Curtis distance as implemented in the microbiome package (version 1.1.10013) [74]. To estimate the variance and dispersion of beta diversity of three experimental factors: “Sponge” (*X. muta*, *A. sventres*), “Depth” (deep, middle and shallow) and “Media type” (MA1/10, M3, OLIGO, GP, Mucin, Crenarchaeota), PERMANOVA was performed with 999 permutations using the adonis and betadisper functions, respectively, as implemented in the vegan package (version 2.5.2) [75]. Furthermore, a heatmap was generated in R Statistical Software using pheatmap package (version 1.0.8) [76] for the overall most abundant OTUs (≥ 0.25% relative abundance, n = 32) across the scraped colonies.

### 4.7. Regrowth and Identification of Picked Isolates

The glycerol stocks of picked colonies were re-grown in 5 mL of the liquid media that were used for their initial isolation. Regrown strains were identified by colony PCR. Briefly, cell lysis was conducted by centrifuging 2 mL of the liquid cultures at 14,000× *g*, and subsequently the obtained pellet was suspended in a sterile PCR tube with 50 µL nuclease-free water (Promega). Furthermore, the cell suspension was stored at −20 °C for 2 h, followed by incubation at 98 °C for 10 min in a PCR thermocycler (BIOKÉ, SensoQuest Gmbh, Goettingen, Germany). The colony identity was determined by amplifying the 16S rRNA gene in a 50 µL PCR reaction mixture containing 32.5 μL nuclease-free water (Promega), 10 μL 5× Phusion Green Buffer (Promega), 1 μL 10 mM dNTPs (Promega), 0.5 μL Phusion HotStart Polymerase (5u/μL, Promega), 1 μL 10 μM forward primer 27F (5′-GTTTGATCCTGGCTCAG-3′) [77], 1 μL 10 μM reverse primer 1492R (5′-GGACTACNVGGGTWTCTAAT-3′) [77], and 1 μL template from the post-lysis cell suspension. The PCR program consisted of initial denaturation at 98 °C for 3 min; 30 cycles of denaturation at 98 °C for 30 s, annealing at 52 °C for 40 s and extension at 72 °C for 90 s, and final extension at 72 °C for 7 min in a PCR thermocyler (BIOKÉ). PCR products were Sanger-sequenced at GATC Biotech (Cologne, Germany) with sequencing primer 806RB [70] to facilitate alignment with the Illumina MiSeq sequencing reads.

## 5. Conclusions

Using six different agar media, we investigated the cultivability of sponge-associated bacteria of the marine sponges *X. muta* and *A. sventres* collected at different depths. Generally, it can be said that the most predominant OTUs recovered from plates from all depths and both sponge species were affiliated to the genera Pseudovibrio, Ruegeria and Microbulbifer. As for the total biomass cultivated from samples of *X. muta*, (but not for *A. sventres*), depth significantly affected the bacteria recovered. However, the impact of depth was less pronounced than the impact of the growth medium and the sponge species targeted. In addition, nearly all isolates recovered in the cultivation experiment did not match the prokaryotes found in the sponges by cultivation-independent means, which reemphasizes that other, more targeted, cultivation conditions are needed to isolate those sponge-associated prokaryotes. Moreover, sponge samples (of the same species) displayed a large variation in numbers of colonies found on agar media, independent of the depth origin and type of media used, which suggests that the viability of prokaryotes in sponge samples in the field or due to treatment and/or storage upon collection may vary and is important to consider for future cultivation experiments.

## Figures and Tables

**Table 1:** Total number of colonies observed from all agar media obtained from individual inocula that were scraped off the plates for sequencing for (**A**) *X. muta* and (**B**) *A. sventres*. XM3–XM15 and AS1–AS10 represent individual specimen of the examined species. The numbers highlighted in bold indicate samples for which 16S rRNA gene amplicon products were obtained and sent for Illumina MiSeq sequencing.

**A. Total number of colonies from *X. muta* samples**											
**Scraping isolates**	***X. muta* lower mesophotic**			***X. muta* upper mesophotic**			***X. muta* shallow**				**Total per medium**
	**XM3**	**XM4**	**XM5**	**XM7**	**XM8**	**XM9**	**XM12**		**XM14**	**XM15**	
MA1/10 agar	**2**	0	**13**	0	**1**	**22**	**1**		0	**1**	40
M3 agar	0	0	0	0	1	0	**43**		**3**	0	47
OLIGO agar	**56**	0	0	**1**	**1**	**82**	0		0	**24**	164
GP agar	0	0	0	1	1	0	**7**		**3**	**0**	12
Mucin agar	**87**	**3**	**12**	**66**	**13**	5	**5**		**1**	**22**	214
Crenarchaeota agar	**61**	**4**	**11**	**51**	**1**	**3**	**2**		0	**40**	173
Total per sample	206	7	36	119	18	112	58		7	87	650
Total per depth	249			249			152				
**B. Total number of colonies from *A. sventres* sample**											
**Scraping isolates**	***A. sventres* upper mesophotic**				***A. sventres* shallow**						**Total per medium**
	**AS1**		**AS2**	**AS3**	**AS6**			**AS7**	**AS10**		
MA1/10 agar	3		0	**2**	**6**			0	0		11
M3 agar	0		0	**4**	1			0	2		7
OLIGO agar	**19**		**513**	**131**	**45**			**69**	**126**		903
GP agar	0		0	0	0			**4**	0		4
Mucin agar	**57**		**377**	**127**	**19**			**53**	**399**		1032
Crenarchaeota agar	**32**		**668**	**130**	**49**			**63**	**125**		1087
Total per sample	111		1558	394	120			189	652		3024
Total per depth	2063				961						

**Table 2:** Multivariate analysis of the influence of sponge species, depth and media type on biomass recovered from plates inoculated with samples of *X. muta* and *A. sventres*. Significant differences are highlighted in bold.

Samples	Parameter	OTUs	df	PERMANOVA		Betadisper	
				*R*^2^	*p*-Value	*F*	*p*-Value
All scraped bacterial colonies (excluding inocula of *X. muta* and *A. sventres*)	Sponge (*X. muta* and *A. sventres*)	371	1	0.10	**0.001**	23.84	**0.001**
	Media type (MA1/10, M3, OLIGO, GP, Mucin, Crenarchaeota)	371	5	0.28	**0.001**	2.43	**0.06**
	Depth (lower mesophotic, upper mesophotic, shallow)	371	2	0.08	**0.003**	0.16	0.84
Scraped bacterial colonies of *X. muta* (excluding inoculum of *X. muta*)	Depth (lower mesophotic, upper mesophotic and shallow)	220	2	0.10	**0.038**	0.84	0.45
	Media type (MA1/10, M3, OLIGO, GP, Mucin, Crenarchaeota)	220	5	0.31	**0.001**	1.20	0.33
Scraped bacterial colonies of *A. sventres* (excluding inoculum of *A. sventres*)	Depth (upper mesophotic and shallow)	151	1	0.06	0.146	0.05	0.83
	Media type (MA1/10, M3, OLIGO, GP, Mucin, Crenarchaeota)	151	5	0.60	**0.001**	4.00	**0.04**

## Supplementary Materials

The following are available online at https://www.mdpi.com/1660-3397/17/10/578/s1, The R markdown file and the required files can be found at https://github.com/mibwurrepo/Indraningrat-et-al.-CultivationSpongedepths2019. Filtered and demultiplexed Illumina MiSeq sequence data can be accessed via the NCBI Sequence Read Archive (SRA) ID PRJNA453745 with accession numbers SRX3998987–SRX3998883.

## References

[1] Mehbub M.F., Lei J., Franco C., Zhang W. (2014). Marine sponge derived natural products between 2001 and 2010: Trends and opportunities for discovery of bioactives. Mar. Drugs.

[2] Sipkema D., Franssen M.C.R., Osinga R., Tramper J., Wijffels R.H. (2005). Marine sponges as pharmacy. Mar. Biotechnol..

[3] Laport M.S., Santos O.C.S., Muricy G. (2009). Marine sponges: Potential sources of new antimicrobial drugs. Curr. Pharm. Biotechnol..

[4] Waters A.L., Peraud O., Kasanah N., Sims J.W., Kothalawala N., Anderson M.A., Abbas S.H., Rao K.V., Jupally V.R., Kelly M. (2014). An analysis of the sponge *Acanthostrongylophora igens*’ microbiome yields an actinomycete that produces the natural product manzamine A. Front. Mar. Sci.

[5] Wilson M.C., Mori T., Ruckert C., Uria A.R., Helf M.J., Takada K., Gernert C., Steffens U.A.E., Heycke N., Schmitt S. (2014). An environmental bacterial taxon with a large and distinct metabolic repertoire. Nature.

[6] Piel J. (2009). Metabolites from symbiotic bacteria. Nat. Prod. Rep..

[7] Taylor M.W., Radax R., Steger D., Wagner M. (2007). Sponge-associated microorganisms: Evolution, ecology, and biotechnological potential. Microbiol. Mol. Biol. R.

[8] Hentschel U., Piel J., Degnan S.M., Taylor M.W. (2012). Genomic insights into the marine sponge microbiome. Nature Rev. Microbiol..

[9] Mohamed N.M., Saito K., Tal Y., Hill R.T. (2010). Diversity of aerobic and anaerobic ammonia-oxidizing bacteria in marine sponges. ISME J..

[10] Hoffmann F., Radax R., Woebken D., Holtappels M., Lavik G., Rapp H.T., Schläppy M.-L., Schleper C., Kuypers M.M.M. (2009). Complex nitrogen cycling in the sponge Geodia barretti. Environ. Microbiol..

[11] Tian R.-M., Wang Y., Bougouffa S., Gao Z.-M., Cai L., Bajic V., Qian P.-Y. (2014). Genomic analysis reveals versatile heterotrophic capacity of a potentially symbiotic sulfur-oxidizing bacterium in sponge. Environ. Microbiol..

[12] Lavy A., Keren R., Yu K., Thomas B.C., Alvarez-Cohen L., Banfield J.F., Ilan M. (2018). A novel Chromatiales bacterium is a potential sulfide oxidizer in multiple orders of marine sponges. Environ. Microbiol..

[13] Zhang F., Blasiak L.C., Karolin J.O., Powell R.J., Geddes C.D., Hill R.T. (2015). Phosphorus sequestration in the form of polyphosphate by microbial symbionts in marine sponges. Proc. Natl. Acad. Sci. USA.

[14] Schippers K.J., Sipkema D., Osinga R., Smidt H., Pomponi S.A., Martens D.E., Wijffels R.H. (2012). Cultivation of sponges, sponge cells and symbionts: Achievements and future prospects. Adv. Mar. Biol..

[15] Joint I., Muhling M., Querellou J. (2010). Culturing marine bacteria—An essential prerequisite for biodiscovery. Microb. Biotechnol..

[16] Sipkema D., Schippers K., Maalcke W.J., Yang Y., Salim S., Blanch H.W. (2011). Multiple approaches to enhance the cultivability of bacteria associated with the marine sponge *Haliclona* (gellius) sp.. Appl. Environ. Microb.

[17] Santos O.C.S., Pontes P.V.M.L., Santos J.F.M., Muricy G., Giambiagi-deMarval M., Laport M.S. (2010). Isolation, characterization and phylogeny of sponge-associated bacteria with antimicrobial activities from Brazil. Res. Microbiol..

[18] Hentschel U., Schmid M., Wagner M., Fieseler L., Gernert C., Hacker J. (2001). Isolation and phylogenetic analysis of bacteria with antimicrobial activities from the Mediterranean sponges *Aplysina aerophoba* and *Aplysina cavernicola*. FEMS Microbiol. Ecol..

[19] Muscholl-Silberhorn A., Thiel V., Imhoff J.F. (2008). Abundance and bioactivity of cultured sponge-associated bacteria from the mediterranean sea. Microb. Ecol..

[20] Matobole R.M., van Zyl L.J., Parker-Nance S., Davies-Coleman M.T., Trindade M. (2017). Antibacterial activities of bacteria isolated from the marine sponges *Isodictya compressa* and *Higginsia bidentifera* collected from Algoa Bay, South Africa. Mar. Drugs.

[21] Subramani R., Sipkema D. (2019). Marine rare Actinomycetes: A promising source of structurally diverse and unique novel natural products. Mar. Drugs..

[22] Indraningrat A.A.G., Smidt H., Sipkema D. (2016). Bioprospecting sponge-associated microbes for antimicrobial compounds. Mar. Drugs.

[23] Olson J.B., Gao X.M. (2013). Characterizing the bacterial associates of three Caribbean sponges along a gradient from shallow to mesophotic depths. FEMS Microbiol. Ecol..

[24] Morrow K.M., Fiore C.L., Lesser M.P. (2016). Environmental drivers of microbial community shifts in the giant barrel sponge, *Xestospongia muta*, over a shallow to mesophotic depth gradient. Environ. Microbiol..

[25] Steinert G., Taylor M.W., Deines P., Simister R.L., de Voogd N.J., Hoggard M., Schupp P.J. (2016). In four shallow and mesophotic tropical reef sponges from Guam the microbial community largely depends on host identity. PeerJ..

[26] Beazley L.I., Kenchington E.L., Murillo F.J., Sacau M.D. (2013). Deep-sea sponge grounds enhance diversity and abundance of epibenthic megafauna in the Northwest Atlantic. ICES J. Mar. Sci.

[27] Thoms C., Schupp P. (2005). Biotechnological potential of marine sponges and their associated bacteria as producers of new pharmaceuticals (part II). J. Int. Biotechnol. Law.

[28] Thomas T., Moitinho-Silva L., Lurgi M., Bjork J.R., Easson C., Astudillo-Garcia C., Olson J.B., Erwin P.M., Lopez-Legentil S., Luter H. (2016). Diversity, structure and convergent evolution of the global sponge microbiome. Nat. Commun..

[29] Olson J.B., Kellogg C.A. (2010). Microbial ecology of corals, sponges, and algae in mesophotic coral environments. FEMS Microbiol. Ecol..

[30] Slattery M., Lesser M.P., Brazeau D., Stokes M.D., Leichter J.J. (2011). Connectivity and stability of mesophotic coral reefs. J. Exp. Mar. Biol. Ecol..

[31] Webster N.S., Taylor M.W. (2012). Marine sponges and their microbial symbionts: Love and other relationships. Environ. Microbiol..

[32] Borchert E., Jackson S.A., O’Gara F., Dobson A.D.W. (2016). Diversity of natural product biosynthetic genes in the microbiome of the deep sea sponges *Inflatella pellicula*, *Poecillastra compressa*, and *Stelletta normani*. Front. Microbiol..

[33] Sipkema D. (2017). Marine biotechnology: Diving deeper for drugs. Microb. Biotechnol..

[34] Parra-Velandia F.J., Zea S., Van Soest R.W.M. (2014). Reef sponges of the genus *Agelas* (Porifera: Demospongiae) from the Greater Caribbean. Zootaxa.

[35] Van Soest R., Meesters E.H.W.G., Becking L.E. (2014). Deep-water sponges (Porifera) from Bonaire and Klein Curaçao, Southern Caribbean. Zootaxa.

[36] Zhou X.F., Xu T.H., Yang X.W., Huang R.M., Yang B., Tang L., Liu Y.H. (2010). Chemical and biological aspects of marine sponges of the genus *Xestospongia*. Chem. Biodiv.

[37] Zhang H.W., Dong M.L., Chen J.W., Wang H., Tenney K., Crews P. (2017). Bioactive secondary metabolites from the marine sponge genus *Agelas*. Mar. Drugs.

[38] Montalvo N.F., Davis J., Vicente J., Pittiglio R., Ravel J., Hill R.T. (2014). Integration of culture-based and molecular analysis of a complex sponge-associated bacterial community. Plos One.

[39] Indraningrat A.A.G., Steinert G., Becking L.E., Mueller B., de Goeij J., Smidt H., Sipkema D. Depth affects sponge prokaryotic communities and their antimicrobial activities in two Demosponges, Xestospongia muta and Agelas sventres.

[40] Versluis D., McPherson K., Passel M., Smidt H., Sipkema D. (2017). Recovery of previously uncultured bacterial genera from three Mediterranean sponges. Mar. Biotechnol..

[41] Hardoim C.C.P., Cardinale M., Cucio A.C.B., Esteves A.I.S., Berg G., Xavier J.R., Cox C.J., Costa R. (2014). Effects of sample handling and cultivation bias on the specificity of bacterial communities in keratose marine sponges. Front. Microbiol..

[42] Graça A.P., Bondoso J., Gaspar H., Xavier J.R., Monteiro M.C., de la Cruz M., Oves-Costales D., Vicente F., Lage O.M. (2013). Antimicrobial activity of heterotrophic bacterial communities from the marine sponge *Erylus discophorus* (Astrophorida, Geodiidae). PLoS ONE.

[43] Chaib De Mares M., Jiménez D.J., Palladino G., Gutleben J., Lebrun L.A., Muller E.E.L., Wilmes P., Sipkema D., van Elsas J.D. (2018). Expressed protein profile of a Tectomicrobium and other microbial symbionts in the marine sponge Aplysina aerophoba as evidenced by metaproteomics. Sci. Rep..

[44] Karimi E., Slaby B.M., Soares A.R., Blom J., Hentschel U., Costa R. (2018). Metagenomic binning reveals versatile nutrient cycling and distinct adaptive features in alphaproteobacterial symbionts of marine sponges. FEMS Microbiol. Ecol..

[45] Karimi E., Ramos M., Gonçalves J.M.S., Xavier J.R., Reis M.P., Costa R. (2017). Comparative metagenomics reveals the distinctive adaptive features of the *Spongia officinalis* endosymbiotic consortium. Front. Microbiol..

[46] Gutleben J., Chaib De Mares M., van Elsas J.D., Smidt H., Overmann J., Sipkema D. (2018). The multi-omics promise in context: From sequence to microbial isolate *Crit*. Rev. Microbiol..

[47] Slaby B.M., Hackl T., Horn H., Bayer K., Hentschel U. (2017). Metagenomic binning of a marine sponge microbiome reveals unity in defense but metabolic specialization. ISME J..

[48] Lai Q., Wang J., Gu L., Zheng T., Shao Z. (2013). *Alcanivorax marinus* sp. nov., isolated from deep-sea water. Int. J. Syst. Evol. Microbiol..

[49] Wang G., Barrett N.H., McCarthy P.J. (2017). Draft genome sequence of deep-sea *Alteromonas* sp. strain V450 isolated from the marine sponge *Leiodermatium* sp.. Genome. Announc.

[50] Kyoung Kwon K., Hye Oh J., Yang S.-H., Seo H.-S., Lee J.-H. (2015). *Alcanivorax gelatiniphagus* sp. nov., a marine bacterium isolated from tidal flat sediments enriched with crude oil. Int. J. Syst. Evol. Microbiol..

[51] Lesser M.P., Slattery M., Leichter J.J. (2009). Ecology of mesophotic coral reefs. J. Exp. Mar. Biol. Ecol..

[52] Liu Q., Fang J., Li J., Zhang L., Xie B.-B., Chen X.-L., Zhang Y.-Z. (2018). Depth-resolved variations of cultivable bacteria and their extracellular enzymes in the water column of the New Britain Trench. Front. Microbiol..

[53] Kai W., Peisheng Y., Rui M., Wenwen J., Zongze S. (2017). Diversity of culturable bacteria in deep-sea water from the South Atlantic Ocean. Bioengineered.

[54] Enticknap J.J., Kelly M., Peraud O., Hill R.T. (2006). Characterization of a culturable Alphaproteobacterial symbiont common to many marine sponges and evidence for vertical transmission via sponge larvae. App. Env. Microbiol..

[55] Romano S. (2018). Ecology and biotechnological potential of bacteria belonging to the genus. Appl. Environ. Microbiol..

[56] Margassery L.M., Kennedy J., O’Gara F., Dobson A.D., Morrissey J.P. (2012). Diversity and antibacterial activity of bacteria isolated from the coastal marine sponges *Amphilectus fucorum* and *Eurypon major*. Lett. App. Microbiol..

[57] Brinkmann C., Kearns P., Evans-Illidge E., Kurtböke D. (2017). Diversity and bioactivity of marine bacteria associated with the sponges *Candidaspongia flabellata* and *Rhopaloeides odorabile* from the Great Barrier Reef in Australia. Diversity.

[58] Mohamed N.M., Cicirelli E.M., Kan J., Chen F., Fuqua C., Hill R.T. (2008). Diversity and quorum-sensing signal production of Proteobacteria associated with marine sponges. Environ. Microbiol..

[59] Zan J., Cicirelli E.M., Mohamed N.M., Sibhatu H., Kroll S., Choi O., Uhlson C.L., Wysoczynski C.L., Murphy R.C., Churchill M.E.A. (2012). A complex LuxR–LuxI type quorum sensing network in a roseobacterial marine sponge symbiont activates flagellar motility and inhibits biofilm formation. Mol. Microbiol..

[60] Quévrain E., Domart-Coulon I., Pernice M., Bourguet-Kondracki M.-L. (2009). Novel natural parabens produced by a *Microbulbifer* bacterium in its calcareous sponge host *Leuconia nivea*. Environ. Microbiol..

[61] Esteves A.I.S., Amer N., Nguyen M., Thomas T. (2016). Sample processing impacts the viability and cultivability of the sponge microbiome. Front. Microbiol..

[62] Munroe S., Martens D.E., Sipkema D., Pomponi S.A. (2018). Comparison of cryopreservation techniques for cells of the marine sponge *Dysidea etheria*. Cryoletters.

[63] Olson J.B., Lord C.C., McCarthy P.J. (2000). Improved Recoverability of Microbial Colonies from Marine Sponge Samples. Microbiol. Ecol..

[64] Zhang H., Zhang W., Jin Y., Jin M., Yu X. (2008). A comparative study on the phylogenetic diversity of culturable actinobacteria isolated from five marine sponge species. Antonie van Leeuwenhoek.

[65] Santavy D.L., Colwell R.R. (1990). Comparison of bacterial communities associated with the Caribbean Sclerosponge *Ceratoporella nicholsoni* and ambient seawater. Mar. Ecol. Prog. Ser..

[66] Olson J.B., McCarthy P.J. (2005). Associated bacterial communities of two deep-water sponges. Aquat. Microb. Ecol..

[67] LibreTexts. https://bio.libretexts.org/Ancillary_Materials/Laboratory_Experiments/Microbiology_Labs/Microbiology_Labs_I/08%3A_Bacterial_Colony_Morphology.

[68] Van Lingen H.J., Edwards J.E., Vaidya J.D., van Gastelen S., Saccenti E., van den Bogert B., Bannink A., Smidt H., Plugge C.M., Dijkstra J. (2017). Diurnal dynamics of gaseous and dissolved metabolites and microbiota composition in the bovine rumen. Front. Microbiol..

[69] Parada A.E., Needham D.M., Fuhrman J.A. (2016). Every base matters: Assessing small subunit rRNA primers for marine microbiomes with mock communities, time series and global field samples. Environ. Microbiol..

[70] Apprill A., McNally S., Parsons R., Weber L. (2015). Minor revision to V4 region SSU rRNA 806R gene primer greatly increases detection of SAR11 bacterioplankton. Aquat. Microb. Ecol..

[71] Ramiro-Garcia J., Hermes G., Giatsis C., Sipkema D., Zoetendal E., Schaap P., Smidt H. (2016). NG-Tax, a highly accurate and validated pipeline for analysis of 16S rRNA amplicons from complex biomes. F1000Research.

[72] Dat T.T.H., Steinert G., Thi Kim Cuc N., Smidt H., Sipkema D. (2018). Archaeal and bacterial diversity and community composition from 18 phylogenetically divergent sponge species in Vietnam. PeerJ..

[73] Yilmaz P., Parfrey L.W., Yarza P., Gerken J., Pruesse E., Quast C., Schweer T., Peplies J., Ludwig W., Glöckner F.O. (2014). The SILVA and “All-species Living Tree Project (LTP)” taxonomic frameworks. Nucleic. Acids. Res.

[74] Lahti L., Shetty A.S., Blake T., Salojarvi J. (2017). Microbiome R Package. https://bioconductor.org/packages/devel/bioc/html/microbiome.html.

[75] Oksanen J., Blanchet F.G., Friendly M., Kindt R., Legendre P., McGlinn D., Minchin P.R., O’Hara R.B., Simpson G.L., Solymos P.M. (2018). Vegan: Community Ecology Package. https://cran.r-project.org/web/packages/vegan/vegan.pdf.

[76] Kolde R. (2015). pheatmap: Pretty Heatmaps. https://cran.r-project.org/web/packages/pheatmap/pheatmap.pdf.

[77] Lane D.J., Stackebrandt E., Goodfellow M. (1991). 16S/23S rRNA Sequencing. Nucleic Acid Techniques in Bacterial Systematic.

