# Supplementary material for: Cultivation of Sponge-Associated Bacteria from Agelas sventres and Xestospongia muta Collected from Different Depths
Source: Mar Drugs. 2019 Oct 11;17(10):578. doi: 10.3390/md17100578 (PMC6836257; doi:10.3390/md17100578)
Supplement: Supplementary file 1 [file marinedrugs-17-00578-s001.pdf]

marine drugs

Supplementary Materials:

Cultivation of sponge-associated bacteria from *Agelas sventres* and *Xestospongia muta* collected from different depths

Anak Agung Gede Indraningrat [1,2,*], Sebastian Micheller [1], Mandy Runderkamp [1], Ina Sauerland [1], Leontine E. Becking [3,4], Hauke Smidt [1] and Detmer Sipkema [1,*]

1 Laboratory of Microbiology, Wageningen University & Research, Stippeneng 4, 6708 WE, Wageningen, The Netherlands
2 Faculty of Medicine and Health Science, Warmadewa University, Jln Terompong 24, 80239, Denpasar, Bali, Indonesia
3 Marine Animal Ecology Group, Wageningen University & Research, Droevendaalsesteeg 1, 6708 PB, Wageningen, The Netherlands
4 Wageningen Marine Research, Wageningen University & Research, Ankerpark 27, 1781 AG, Den Helder, The Netherlands
* Correspondence: indraningrat@warmadewa.ac.id (A.A.G.I.); detmer.sipkema@wur.nl (D.S.)

Table of Content

Supplementary Figure 1. Sponge specimens from different depths that were used in this study

Table S1. Reads Distribution of sponge incoculums and scraped bacterial communities on agar plates.

Table S2. Distribution of OTUs that specifically found in sponges from each depth category.

Table S3. Number of colonies counted from each sponge per agar medium from agar plates dedicated for picking

Table S4. Colonies picked from *X. muta* and *A. sventres* with their Colony Morphology Code (CMC), clades number or singleton (based on 16S rRNA gene sequence).

Table S5. Sponge specimens that were used as inocula for cultivation.

Table S6. List of barcodes, linker, unitag sequences and accession numbers of sponge inoculums and scraped bacterial communities.

marine drugs

MDPI

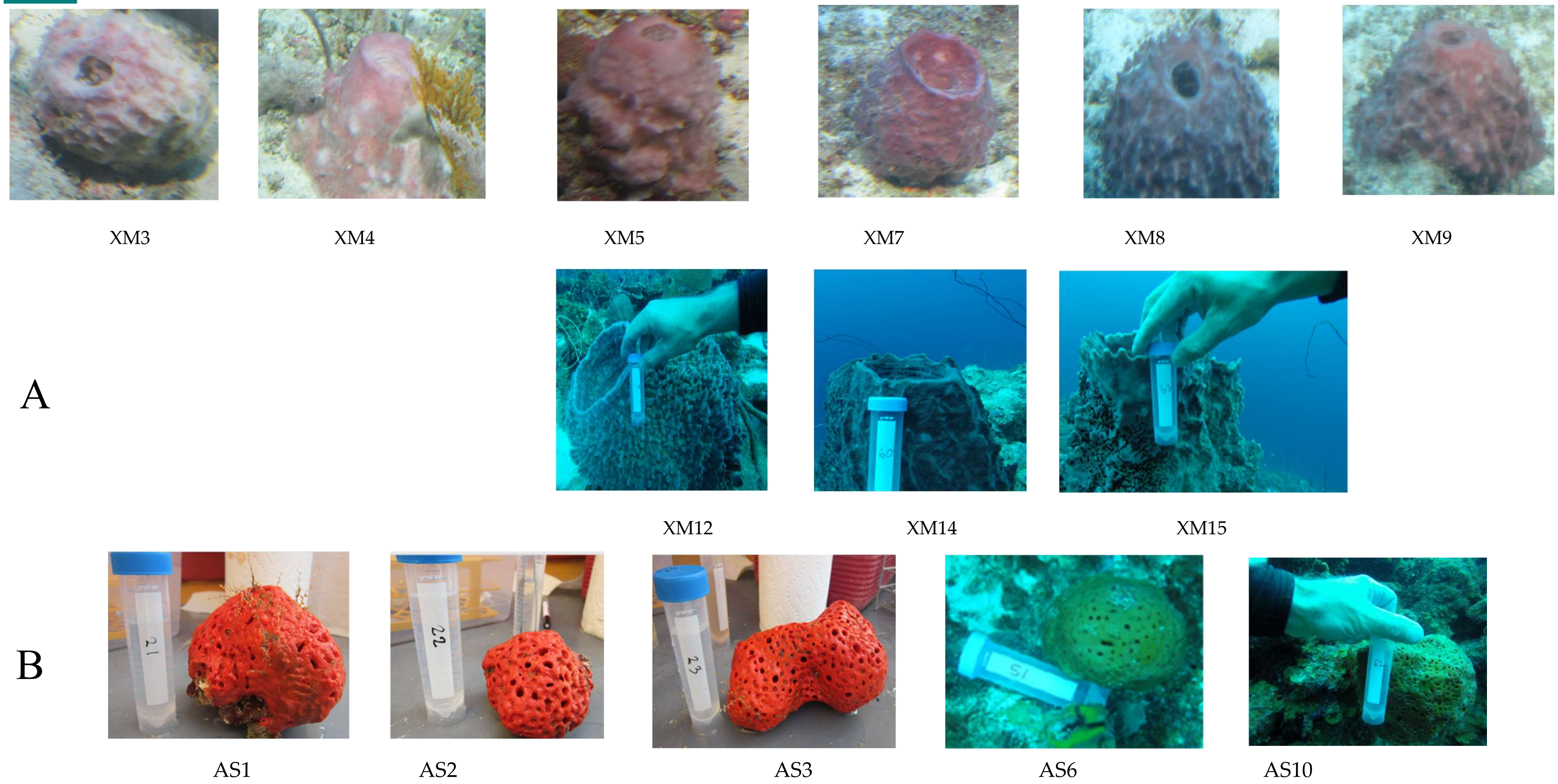

Supplementary Figure 1. Sponge specimens from different depths that were used in this study. Panel A represents underwater figures of nine specimens of *X. muta* collected from different depths: lower mesophotic (XM3 – XM5), upper mesophotic (XM7-XM9), shallow (XM12, XM14, XM15). Panel B shows figures of *A. sventres* individuals collected from upper mesophotic (above water, AS1 – AS3) and shallow depth (underwater, AS6 and AS10). No picture is available for individual AS7. .

marine drugs

MDPI

Table S1. Reads Distribution of sponge inoculums and scraped bacterial communities on agar plates. Panel A is information of *X. muta* and Panel B is information of *A. sventres*. The first initial before the dot symbol indicated the source of samples e.g. Co is for cryostock (sponge inoculum) and MA1/10 indicates Marine Agar 1/10. The observed total OTUs indicated total OTUs found in samples (including overlapping with OTUs found in other samples).

A. Reads information of *X. muta*

Sample	Reads	Observed total OTU(s)	Depth category	Depth (m)	Origin
Co.XM3	78776	96	Lower mesophotic	72	sponge inoculum
Co.XM4	158378	123	Lower mesophotic	85	sponge inoculum
Co.XM5	99149	126	Lower mesophotic	82	sponge inoculum
MA1/10.XM3	146456	6	Lower mesophotic	72	MA1/10 agar
MA1/10.XM5	73920	2	Lower mesophotic	82	MA1/10 agar
OLIGO.XM3	77765	35	Lower mesophotic	72	OLIGO agar
CR.XM3	43756	12	Lower mesophotic	72	Crenarchaeota agar
CR.XM4	93884	22	Lower mesophotic	85	Crenarchaeota agar
CR.XM5	92864	8	Lower mesophotic	82	Crenarchaeota agar
Mucin.XM3	27617	18	Lower mesophotic	72	Mucin agar
Mucin.XM4	14139	23	Lower mesophotic	85	Mucin agar
Mucin.XM5	39629	10	Lower mesophotic	82	Mucin agar
Co.XM7	41546	132	Upper mesophotic	52	sponge inoculum
Co.XM8	148791	107	Upper mesophotic	52	sponge inoculum
Co.XM9	161949	118	Upper mesophotic	48	sponge inoculum
GP.XM7	114998	3	Upper mesophotic	52	GP agar
MA1/10.XM8	103339	1	Upper mesophotic	52	MA1/10 agar
MA1/10.XM9	113685	12	Upper mesophotic	48	MA1/10 agar
OLIGO.XM7	123663	1	Upper mesophotic	52	OLIGO agar
OLIGO.XM8	66328	2	Upper mesophotic	52	OLIGO agar
OLIGO.XM9	76111	2	Upper mesophotic	48	OLIGO agar
CR.XM7	32917	19	Upper mesophotic	52	Crenarchaeota agar
CR.XM8	67533	3	Upper mesophotic	52	Crenarchaeota agar
CR.XM9	123438	10	Upper mesophotic	48	Crenarchaeota agar
Mucin.XM7	65877	19	Upper mesophotic	52	Mucin agar
Mucin.XM8	134209	25	Upper mesophotic	52	Mucin agar
Mucin.XM9	178291	21	Upper mesophotic	48	Mucin agar
Co.XM12	213905	87	Shallow	27	sponge inoculum
Co.XM14	98479	129	Shallow	27	sponge inoculum
Co.XM15	167232	91	Shallow	27	sponge inoculum
GP.XM12	30925	12	Shallow	27	GP agar
GP.XM14	94817	2	Shallow	27	GP agar

GP.XM15	78409	5	Shallow	27	GP agar
MA1/10.XM12	91988	3	Shallow	27	MA1/10 agar
MA1/10.XM15	66874	2	Shallow	27	MA1/10 agar
CR.XM12	176874	3	Shallow	27	Crenarchaeota agar
CR.XM15	156966	30	Shallow	27	Crenarchaeota agar
M3.XM12	208300	21	Shallow	27	M3 agar
M3.XM14	87090	2	Shallow	27	M3 agar
OL.XM15	51402	24	Shallow	27	OLIGO agar
Mucin.XM12	98191	21	Shallow	27	Mucin agar
Mucin.XM14	67221	3	Shallow	27	Mucin agar
Mucin.XM15	130345	22	Shallow	27	Mucin agar

B. Reads information of *A.sventres*

Sample	Reads	Obseved OTU(s)	Depth category	Depth (m)	Origin
Co.AS1	21866	52	Upper mesophotic	54	sponge inoculum
Co.AS2	116122	76	Upper mesophotic	52	sponge inoculum
Co.AS3	121073	63	Upper mesophotic	52	sponge inoculum
OL.AS1	29723	20	Upper mesophotic	54	OLIGO agar
OL.AS2	65569	19	Upper mesophotic	52	OLIGO agar
OL.AS3	38591	35	Upper mesophotic	52	OLIGO agar
CR.AS1	25919	29	Upper mesophotic	54	Crenarchaeota agar
CR.AS2	15059	19	Upper mesophotic	52	Crenarchaeota agar
CR.AS3	28396	37	Upper mesophotic	52	Crenarchaeota agar
Mucin.AS1	60989	30	Upper mesophotic	54	Mucin agar
Mucin.AS2	22471	18	Upper mesophotic	52	Mucin agar
Mucin.AS3	9433	33	Upper mesophotic	52	Mucin agar
MA1/10.AS3	70371	8	Upper mesophotic	52	MA1/10 agar
M3.AS3	44351	23	Upper mesophotic	52	M3 agar
Co.AS6	106056	58	Shallow	12	sponge inoculum
Co.AS7	39995	55	Shallow	12	sponge inoculum
Co.AS10	61242	67	Shallow	27	sponge inoculum
OLIGO.AS6	24303	34	Shallow	12	OLIGO agar
OLIGO.AS7	32296	42	Shallow	12	OLIGO agar
OLIGO.AS10	44682	39	Shallow	27	OLIGO agar
CR.AS6	48877	38	Shallow	12	Crenarchaeota agar
CR.AS7	9846	37	Shallow	12	Crenarchaeota agar
CR.AS10	18231	34	Shallow	27	Crenarchaeota agar
Mucin.AS6	2360	22	Shallow	12	Mucin agar
Mucin.AS7	13087	19	Shallow	12	Mucin agar
Mucin.AS10	25977	28	Shallow	27	Mucin agar

MA1/10.AS6	32873	20	Shallow	12	MA1/10 agar
GP.AS7	97963	8	Shallow	12	GP agar

marine drugs
MDPI

Table S2. Distribution of OTUs that specifically found in sponge from each depth category.

Sponge	Criteria	OTU ID	NG-TAX annotation
X. muta	OTUs present in scraped biomass of lower mesophotic *X. muta*	OTU1296	k__Bacteria; p__Actinobacteria; c__Actinobacteria; o__Corynebacteriales; f__Mycobacteriaceae; g__*Mycobacterium*
		OTU1364	k__Bacteria; p__Bacteroidetes; c__Flavobacteriia; o__Flavobacteriales; f__Flavobacteriaceae; g__*Aureivirga*
		OTU1356	k__Bacteria; p__Bacteroidetes; c__Flavobacteriia; o__Flavobacteriales; f__Flavobacteriaceae; g__*Salegentibacter*
		OTU1362	k__Bacteria; p__Bacteroidetes; c__Flavobacteriia; o__Flavobacteriales; f__Flavobacteriaceae; g__*Salegentibacter*
		OTU1311	k__Bacteria; p__Bacteroidetes; c__Flavobacteriia; o__Flavobacteriales; f__Flavobacteriaceae; g__*Tenacibaculum*
		OTU1312	k__Bacteria; p__Bacteroidetes; c__Flavobacteriia; o__Flavobacteriales; f__Flavobacteriaceae; g__*Tenacibaculum*
		OTU1316	k__Bacteria; p__Bacteroidetes; c__Flavobacteriia; o__Flavobacteriales; f__Flavobacteriaceae; g__*Tenacibaculum*
		OTU1361	k__Bacteria; p__Firmicutes; c__Bacilli; o__Bacillales; f__Bacillaceae
		OTU1377	k__Bacteria; p__Firmicutes; c__Bacilli; o__Bacillales; f__Bacillaceae
		OTU1382	k__Bacteria; p__Firmicutes; c__Bacilli; o__Bacillales; f__Bacillaceae

		OTU1357	k__Bacteria; p__Firmicutes; c__Bacilli; o__Bacillales; f__Bacillaceae; g__*Fictibacillus*
		OTU1360	k__Bacteria; p__Firmicutes; c__Bacilli; o__Bacillales; f__Bacillaceae; g__*Fictibacillus*
		OTU1363	k__Bacteria; p__Firmicutes; c__Bacilli; o__Bacillales; f__Bacillaceae; g__*Fictibacillus*
		OTU1367	k__Bacteria; p__Firmicutes; c__Bacilli; o__Bacillales; f__Bacillaceae; g__*Marinococcus*
		OTU1368	k__Bacteria; p__Firmicutes; c__Bacilli; o__Bacillales; f__Bacillaceae; g__*Marinococcus*
		OTU1369	k__Bacteria; p__Firmicutes; c__Bacilli; o__Bacillales; f__Bacillaceae; g__*Marinococcus*
		OTU1370	k__Bacteria; p__Firmicutes; c__Bacilli; o__Bacillales; f__Bacillaceae; g__*Marinococcus*
		OTU1371	k__Bacteria; p__Firmicutes; c__Bacilli; o__Bacillales; f__Bacillaceae; g__*Marinococcus*
		OTU1373	k__Bacteria; p__Firmicutes; c__Bacilli; o__Bacillales; f__Bacillaceae; g__*Marinococcus*
		OTU1374	k__Bacteria; p__Firmicutes; c__Bacilli; o__Bacillales; f__Bacillaceae; g__*Marinococcus*
		OTU1376	k__Bacteria; p__Firmicutes; c__Bacilli; o__Bacillales; f__Bacillaceae; g__*Marinococcus*

		OTU1378	k__Bacteria; p__Firmicutes; c__Bacilli; o__Bacillales; f__Bacillaceae; g__*Marinococcus*
		OTU1381	k__Bacteria; p__Firmicutes; c__Bacilli; o__Bacillales; f__Bacillaceae; g__*Marinococcus*
		OTU1383	k__Bacteria; p__Firmicutes; c__Bacilli; o__Bacillales; f__Bacillaceae; g__*Marinococcus*
		OTU1221	k__Bacteria; p__Firmicutes; c__Bacilli; o__Bacillales; f__Planococcaceae
		OTU1222	k__Bacteria; p__Firmicutes; c__Bacilli; o__Bacillales; f__Planococcaceae
		OTU1251	k__Bacteria; p__Proteobacteria; c__Alphaproteobacteria; o__Rhodobacterales; f__Rhodobacteraceae
		OTU1256	k__Bacteria; p__Proteobacteria; c__Alphaproteobacteria; o__Rhodobacterales; f__Rhodobacteraceae; g__
		OTU1293	k__Bacteria; p__Proteobacteria; c__Alphaproteobacteria; o__Rhodobacterales; f__Rhodobacteraceae
		OTU1302	k__Bacteria; p__Proteobacteria; c__Alphaproteobacteria; o__Rhodobacterales; f__Rhodobacteraceae
		OTU1310	k__Bacteria; p__Proteobacteria; c__Alphaproteobacteria; o__Rhodobacterales; f__Rhodobacteraceae
		OTU1313	k__Bacteria; p__Proteobacteria; c__Alphaproteobacteria; o__Rhodobacterales; f__Rhodobacteraceae

		OTU1314	k__Bacteria; p__Proteobacteria; c__Alphaproteobacteria; o__Rhodobacterales; f__Rhodobacteraceae
		OTU1315	k__Bacteria; p__Proteobacteria; c__Alphaproteobacteria; o__Rhodobacterales; f__Rhodobacteraceae
		OTU1375	k__Bacteria; p__Proteobacteria; c__Alphaproteobacteria; o__Rhodobacterales; f__Rhodobacteraceae
		OTU1379	k__Bacteria; p__Proteobacteria; c__Alphaproteobacteria; o__Rhodobacterales; f__Rhodobacteraceae
		OTU1380	k__Bacteria; p__Proteobacteria; c__Alphaproteobacteria; o__Rhodobacterales; f__Rhodobacteraceae
		OTU1304	k__Bacteria; p__Proteobacteria; c__Alphaproteobacteria; o__Rhodobacterales; f__Rhodobacteraceae; g__*Mameliella*
		OTU1289	k__Bacteria; p__Proteobacteria; c__Alphaproteobacteria; o__Rhodobacterales; f__Rhodobacteraceae; g__uncultured
		OTU1217	k__Bacteria; p__Proteobacteria; c__Alphaproteobacteria; o__Rhodospirillales; f__Acetobacteraceae; g__*Roseomonas*
		OTU1218	k__Bacteria; p__Proteobacteria; c__Alphaproteobacteria; o__Rhodospirillales; f__Acetobacteraceae; g__*Roseomonas*
		OTU1219	k__Bacteria; p__Proteobacteria; c__Alphaproteobacteria; o__Rhodospirillales; f__Acetobacteraceae; g__*Roseomonas*
		OTU1295	k__Bacteria; p__Proteobacteria; c__Alphaproteobacteria; o__Sphingomonadales; f__Erythrobacteraceae; g__*Erythrobacter*

		OTU1300	k__Bacteria; p__Proteobacteria; c__Alphaproteobacteria; o__Sphingomonadales; f__Erythrobacteraceae; g__*Erythrobacter*
		OTU1301	k__Bacteria; p__Proteobacteria; c__Alphaproteobacteria; o__Sphingomonadales; f__Erythrobacteraceae; g__*Erythrobacter*
		OTU1307	k__Bacteria; p__Proteobacteria; c__Alphaproteobacteria; o__Sphingomonadales; f__Erythrobacteraceae; g__*Erythrobacter*
		OTU1372	k__Bacteria; p__Proteobacteria; c__Alphaproteobacteria; o__Sphingomonadales; f__Erythrobacteraceae; g__*Erythrobacter*
		OTU1384	k__Bacteria; p__Proteobacteria; c__Alphaproteobacteria; o__Sphingomonadales; f__Erythrobacteraceae; g__*Erythrobacter*
		OTU1385	k__Bacteria; p__Proteobacteria; c__Alphaproteobacteria; o__Sphingomonadales; f__Erythrobacteraceae; g__*Erythrobacter*
		OTU1386	k__Bacteria; p__Proteobacteria; c__Alphaproteobacteria; o__Sphingomonadales; f__Erythrobacteraceae; g__*Erythrobacter*
		OTU1387	k__Bacteria; p__Proteobacteria; c__Alphaproteobacteria; o__Sphingomonadales; f__Erythrobacteraceae; g__*Erythrobacter*
		OTU1388	k__Bacteria; p__Proteobacteria; c__Alphaproteobacteria; o__Sphingomonadales; f__Erythrobacteraceae; g__*Erythrobacter*
		OTU1303	k__Bacteria; p__Proteobacteria; c__Gammaproteobacteria
		OTU864	k__Bacteria; p__Proteobacteria; c__Gammaproteobacteria; o__Alteromonadales; f__Alteromonadaceae; g__*Alteromonas*
		OTU1359	k__Bacteria; p__Proteobacteria; c__Gammaproteobacteria; o__Alteromonadales; f__Alteromonadaceae; g__*Alteromonas*

		OTU1365	k__Bacteria; p__Proteobacteria; c__Gammaproteobacteria; o__Alteromonadales; f__Alteromonadaceae; g__*Alteromonas*
		OTU1292	k__Bacteria; p__Proteobacteria; c__Gammaproteobacteria; o__Cellvibrionales; f__Halieaceae
		OTU1235	k__Bacteria; p__Proteobacteria; c__Gammaproteobacteria; o__Oceanospirillales; f__Alcanivoracaceae; g__*Alcanivorax*
		OTU1236	k__Bacteria; p__Proteobacteria; c__Gammaproteobacteria; o__Oceanospirillales; f__Alcanivoracaceae; g__*Alcanivorax*
		OTU1243	k__Bacteria; p__Proteobacteria; c__Gammaproteobacteria; o__Oceanospirillales; f__Alcanivoracaceae; g__*Alcanivorax*
		OTU1244	k__Bacteria; p__Proteobacteria; c__Gammaproteobacteria; o__Oceanospirillales; f__Alcanivoracaceae; g__*Alcanivorax*
		OTU1246	k__Bacteria; p__Proteobacteria; c__Gammaproteobacteria; o__Oceanospirillales; f__Alcanivoracaceae; g__*Alcanivorax*
		OTU1248	k__Bacteria; p__Proteobacteria; c__Gammaproteobacteria; o__Oceanospirillales; f__Alcanivoracaceae; g__*Alcanivorax*
		OTU1250	k__Bacteria; p__Proteobacteria; c__Gammaproteobacteria; o__Oceanospirillales; f__Alcanivoracaceae; g__*Alcanivorax*
		OTU1252	k__Bacteria; p__Proteobacteria; c__Gammaproteobacteria; o__Oceanospirillales; f__Alcanivoracaceae; g__*Alcanivorax*
		OTU1253	k__Bacteria; p__Proteobacteria; c__Gammaproteobacteria; o__Oceanospirillales; f__Alcanivoracaceae; g__*Alcanivorax*

		OTU1258	k__Bacteria; p__Proteobacteria; c__Gammaproteobacteria; o__Oceanospirillales; f__Alcanivoracaceae; g__*Alcanivorax*
		OTU1260	k__Bacteria; p__Proteobacteria; c__Gammaproteobacteria; o__Oceanospirillales; f__Alcanivoracaceae; g__*Alcanivorax*
		OTU1261	k__Bacteria; p__Proteobacteria; c__Gammaproteobacteria; o__Oceanospirillales; f__Alcanivoracaceae; g__*Alcanivorax*
		OTU1262	k__Bacteria; p__Proteobacteria; c__Gammaproteobacteria; o__Oceanospirillales; f__Alcanivoracaceae; g__*Alcanivorax*
		OTU1263	k__Bacteria; p__Proteobacteria; c__Gammaproteobacteria; o__Oceanospirillales; f__Alcanivoracaceae; g__*Alcanivorax*
		OTU1266	k__Bacteria; p__Proteobacteria; c__Gammaproteobacteria; o__Oceanospirillales; f__Alcanivoracaceae; g__*Alcanivorax*
		OTU1290	k__Bacteria; p__Proteobacteria; c__Gammaproteobacteria; o__Oceanospirillales; f__Alcanivoracaceae; g__*Alcanivorax*
		OTU1291	k__Bacteria; p__Proteobacteria; c__Gammaproteobacteria; o__Oceanospirillales; f__Alcanivoracaceae; g__*Alcanivorax*
		OTU1259	k__Bacteria; p__Proteobacteria; c__Gammaproteobacteria; o__Oceanospirillales; f__Alcanivoracaceae; g__*Kangiella*
		OTU1254	k__Bacteria; p__Proteobacteria; c__Gammaproteobacteria; o__Oceanospirillales; f__Hahellaceae; g__*Endozoicomonas*
		OTU1264	k__Bacteria; p__Proteobacteria; c__Gammaproteobacteria; o__Oceanospirillales; f__Hahellaceae; g__*Endozoicomonas*

		OTU1294	k__Bacteria; p__Proteobacteria; c__Gammaproteobacteria; o__Oceanospirillales; f__Halomonadaceae; g__*Halomonas*
		OTU1297	k__Bacteria; p__Proteobacteria; c__Gammaproteobacteria; o__Oceanospirillales; f__Halomonadaceae; g__*Halomonas*
		OTU1298	k__Bacteria; p__Proteobacteria; c__Gammaproteobacteria; o__Oceanospirillales; f__Halomonadaceae; g__*Halomonas*
		OTU1299	k__Bacteria; p__Proteobacteria; c__Gammaproteobacteria; o__Oceanospirillales; f__Halomonadaceae; g__*Halomonas*
		OTU1305	k__Bacteria; p__Proteobacteria; c__Gammaproteobacteria; o__Oceanospirillales; f__Halomonadaceae; g__*Halomonas*
		OTU1306	k__Bacteria; p__Proteobacteria; c__Gammaproteobacteria; o__Oceanospirillales; f__Halomonadaceae; g__*Halomonas*
		OTU1308	k__Bacteria; p__Proteobacteria; c__Gammaproteobacteria; o__Oceanospirillales; f__Halomonadaceae; g__*Halomonas*
		OTU1309	k__Bacteria; p__Proteobacteria; c__Gammaproteobacteria; o__Oceanospirillales; f__Halomonadaceae; g__*Halomonas*
		OTU820	k__Bacteria; p__Proteobacteria; c__Gammaproteobacteria; o__Vibrionales; f__Vibrionaceae; g__*Vibrio*
		OTU1220	k__Bacteria; p__Proteobacteria; c__Gammaproteobacteria; o__Xanthomonadales; f__Xanthomonadaceae; g__*Stenotrophomonas*
	OTUs present in scraped colonies of upper mesophotic *X. muta*	OTU1225	k__Bacteria; p__Actinobacteria; c__Actinobacteria; o__Micrococcales; f__Micrococcaceae

		OTU1231	k__Bacteria; p__Actinobacteria; c__Actinobacteria; o__Micrococcales; f__Micrococcaceae; g__*Micrococcus*
		OTU1232	k__Bacteria; p__Actinobacteria; c__Actinobacteria; o__Micrococcales; f__Micrococcaceae; g__*Micrococcus*
		OTU1393	k__Bacteria; p__Bacteroidetes; c__Flavobacteriia; o__Flavobacteriales; f__Flavobacteriaceae; g__*Aureivirga*
		OTU1322	k__Bacteria; p__Firmicutes; c__Bacilli; o__Bacillales; f__Bacillaceae; g__*Bacillus*
		OTU1394	k__Bacteria; p__Firmicutes; c__Bacilli; o__Bacillales; f__Bacillaceae; g__*Bacillus*
		OTU1267	k__Bacteria; p__Firmicutes; c__Bacilli; o__Bacillales; f__Staphylococcaceae; g__*Staphylococcus*
		OTU1226	k__Bacteria; p__Firmicutes; c__Bacilli; o__Lactobacillales; f__Lactobacillaceae; g__*Pediococcus*
		OTU1227	k__Bacteria; p__Firmicutes; c__Bacilli; o__Lactobacillales; f__Lactobacillaceae; g__*Pediococcus*
		OTU1229	k__Bacteria; p__Firmicutes; c__Bacilli; o__Lactobacillales; f__Lactobacillaceae; g__*Pediococcus*
		OTU1230	k__Bacteria; p__Firmicutes; c__Bacilli; o__Lactobacillales; f__Lactobacillaceae; g__*Pediococcus*
		OTU1390	k__Bacteria; p__Proteobacteria; c__Alphaproteobacteria
		OTU1399	k__Bacteria; p__Proteobacteria; c__Alphaproteobacteria; o__Caulobacterales; f__Caulobacteraceae

		OTU1401	k__Bacteria; p__Proteobacteria; c__Alphaproteobacteria; o__Caulobacterales; f__Caulobacteraceae; g__*Brevundimonas*
		OTU1392	k__Bacteria; p__Proteobacteria; c__Alphaproteobacteria; o__Rhodobacterales; f__Rhodobacteraceae
		OTU1397	k__Bacteria; p__Proteobacteria; c__Alphaproteobacteria; o__Rhodobacterales; f__Rhodobacteraceae
		OTU1317	k__Bacteria; p__Proteobacteria; c__Alphaproteobacteria; o__Rhodobacterales; f__Rhodobacteraceae; g__*Labrenzia*
		OTU1405	k__Bacteria; p__Proteobacteria; c__Alphaproteobacteria; o__Rhodobacterales; f__Rhodobacteraceae; g__*Paracoccus*
		OTU1389	k__Bacteria; p__Proteobacteria; c__Alphaproteobacteria; o__Rhodobacterales; f__Rhodobacteraceae; g__*Pseudovibrio*
		OTU1398	k__Bacteria; p__Proteobacteria; c__Alphaproteobacteria; o__Rhodobacterales; f__Rhodobacteraceae; g__*Pseudovibrio*
		OTU1413	k__Bacteria; p__Proteobacteria; c__Alphaproteobacteria; o__Rhodobacterales; f__Rhodobacteraceae; g__*Pseudovibrio*
		OTU1403	k__Bacteria; p__Proteobacteria; c__Gammaproteobacteria; o__Cellvibrionales; f__Microbulbiferaceae; g__*Microbulbifer*
		OTU1406	k__Bacteria; p__Proteobacteria; c__Gammaproteobacteria; o__Cellvibrionales; f__Microbulbiferaceae; g__*Microbulbifer*
		OTU1407	k__Bacteria; p__Proteobacteria; c__Gammaproteobacteria; o__Cellvibrionales; f__Microbulbiferaceae; g__*Microbulbifer*

		OTU1408	k__Bacteria; p__Proteobacteria; c__Gammaproteobacteria; o__Cellvibrionales; f__Microbulbiferaceae; g__*Microbulbifer*
		OTU1409	k__Bacteria; p__Proteobacteria; c__Gammaproteobacteria; o__Cellvibrionales; f__Microbulbiferaceae; g__*Microbulbifer*
		OTU1410	k__Bacteria; p__Proteobacteria; c__Gammaproteobacteria; o__Cellvibrionales; f__Microbulbiferaceae; g__*Microbulbifer*
		OTU1411	k__Bacteria; p__Proteobacteria; c__Gammaproteobacteria; o__Cellvibrionales; f__Microbulbiferaceae; g__*Microbulbifer*
		OTU1412	k__Bacteria; p__Proteobacteria; c__Gammaproteobacteria; o__Cellvibrionales; f__Microbulbiferaceae; g__*Microbulbifer*
		OTU1402	k__Bacteria; p__Proteobacteria; c__Gammaproteobacteria; o__Oceanospirillales; f__Hahellaceae
		OTU1395	k__Bacteria; p__Proteobacteria; c__Gammaproteobacteria; o__Oceanospirillales; f__Hahellaceae; g__*Endozoicomonas*
		OTU1396	k__Bacteria; p__Proteobacteria; c__Gammaproteobacteria; o__Oceanospirillales; f__Hahellaceae; g__*Endozoicomonas*
		OTU1400	k__Bacteria; p__Proteobacteria; c__Gammaproteobacteria; o__Oceanospirillales; f__Hahellaceae; g__*Endozoicomonas*
	OTUs present in scraped colonies of shallow *X. muta*	OTU1424	k__Bacteria; p__Actinobacteria; c__Actinobacteria; o__Micrococcales; f__Dermabacteraceae; g__*Brachybacterium*
		OTU1425	k__Bacteria; p__Actinobacteria; c__Actinobacteria; o__Micrococcales; f__Dermabacteraceae; g__*Brachybacterium*

		OTU1341	k__Bacteria; p__Actinobacteria; c__Actinobacteria; o__Micrococcales; f__Micrococcaceae
		OTU1344	k__Bacteria; p__Actinobacteria; c__Actinobacteria; o__Micrococcales; f__Micrococcaceae
		OTU1345	k__Bacteria; p__Actinobacteria; c__Actinobacteria; o__Micrococcales; f__Micrococcaceae
		OTU1350	k__Bacteria; p__Actinobacteria; c__Actinobacteria; o__Micrococcales; f__Micrococcaceae
		OTU1354	k__Bacteria; p__Actinobacteria; c__Actinobacteria; o__Micrococcales; f__Micrococcaceae
		OTU1351	k__Bacteria; p__Actinobacteria; c__Actinobacteria; o__Micrococcales; f__Micrococcaceae; g__*Arthrobacter*
		OTU1339	k__Bacteria; p__Actinobacteria; c__Actinobacteria; o__Micrococcales; f__Micrococcaceae; g__*Kocuria*
		OTU1340	k__Bacteria; p__Actinobacteria; c__Actinobacteria; o__Micrococcales; f__Micrococcaceae; g__*Micrococcus*
		OTU1342	k__Bacteria; p__Actinobacteria; c__Actinobacteria; o__Micrococcales; f__Micrococcaceae; g__*Micrococcus*
		OTU1346	k__Bacteria; p__Actinobacteria; c__Actinobacteria; o__Micrococcales; f__Micrococcaceae; g__*Micrococcus*
		OTU1349	k__Bacteria; p__Actinobacteria; c__Actinobacteria; o__Micrococcales; f__Micrococcaceae; g__*Micrococcus*

		OTU1352	k__Bacteria; p__Actinobacteria; c__Actinobacteria; o__Micrococcales; f__Micrococcaceae; g__*Micrococcus*
		OTU1353	k__Bacteria; p__Actinobacteria; c__Actinobacteria; o__Micrococcales; f__Micrococcaceae; g__*Micrococcus*
		OTU1355	k__Bacteria; p__Actinobacteria; c__Actinobacteria; o__Micrococcales; f__Micrococcaceae; g__*Micrococcus*
		OTU1215	k__Bacteria; p__Actinobacteria; c__Actinobacteria; o__Micrococcales; f__Micrococcaceae; g__*Rothia*
		OTU1327	k__Bacteria; p__Firmicutes; c__Bacilli; o__Bacillales
		OTU1329	k__Bacteria; p__Firmicutes; c__Bacilli; o__Bacillales
		OTU1330	k__Bacteria; p__Firmicutes; c__Bacilli; o__Bacillales
		OTU1334	k__Bacteria; p__Firmicutes; c__Bacilli; o__Bacillales; f__Family_XII; g__*Exiguobacterium*
		OTU1204	k__Bacteria; p__Firmicutes; c__Bacilli; o__Bacillales; f__Staphylococcaceae; g__*Staphylococcus*
		OTU1207	k__Bacteria; p__Firmicutes; c__Bacilli; o__Bacillales; f__Staphylococcaceae; g__*Staphylococcus*
		OTU1208	k__Bacteria; p__Firmicutes; c__Bacilli; o__Bacillales; f__Staphylococcaceae; g__*Staphylococcus*
		OTU1210	k__Bacteria; p__Firmicutes; c__Bacilli; o__Bacillales; f__Staphylococcaceae; g__*Staphylococcus*
		OTU1212	k__Bacteria; p__Firmicutes; c__Bacilli; o__Bacillales; f__Staphylococcaceae; g__*Staphylococcus*

		OTU1337	k__Bacteria; p__Firmicutes; c__Bacilli; o__Bacillales; f__Staphylococcaceae; g__*Staphylococcus*
		OTU1338	k__Bacteria; p__Firmicutes; c__Bacilli; o__Bacillales; f__Staphylococcaceae; g__*Staphylococcus*
		OTU1343	k__Bacteria; p__Firmicutes; c__Bacilli; o__Bacillales; f__Staphylococcaceae; g__*Staphylococcus*
		OTU1347	k__Bacteria; p__Firmicutes; c__Bacilli; o__Bacillales; f__Staphylococcaceae; g__*Staphylococcus*
		OTU1348	k__Bacteria; p__Firmicutes; c__Bacilli; o__Bacillales; f__Staphylococcaceae; g__*Staphylococcus*
		OTU1203	k__Bacteria; p__Firmicutes; c__Bacilli; o__Lactobacillales; f__Lactobacillaceae; g__*Lactobacillus*
		OTU1205	k__Bacteria; p__Firmicutes; c__Bacilli; o__Lactobacillales; f__Lactobacillaceae; g__*Lactobacillus*
		OTU1206	k__Bacteria; p__Firmicutes; c__Bacilli; o__Lactobacillales; f__Lactobacillaceae; g__*Lactobacillus*
		OTU1209	k__Bacteria; p__Firmicutes; c__Bacilli; o__Lactobacillales; f__Lactobacillaceae; g__*Pediococcus*
		OTU1211	k__Bacteria; p__Firmicutes; c__Bacilli; o__Lactobacillales; f__Lactobacillaceae; g__*Pediococcus*
		OTU1282	k__Bacteria; p__Proteobacteria; c__Alphaproteobacteria
		OTU1280	k__Bacteria; p__Proteobacteria; c__Alphaproteobacteria; o__Rhodobacterales; f__Rhodobacteraceae

		OTU1287	k__Bacteria; p__Proteobacteria; c__Alphaproteobacteria; o__Rhodobacterales; f__Rhodobacteraceae
		OTU1328	k__Bacteria; p__Proteobacteria; c__Alphaproteobacteria; o__Rhodobacterales; f__Rhodobacteraceae
		OTU1333	k__Bacteria; p__Proteobacteria; c__Alphaproteobacteria; o__Rhodobacterales; f__Rhodobacteraceae
		OTU1415	k__Bacteria; p__Proteobacteria; c__Alphaproteobacteria; o__Rhodobacterales; f__Rhodobacteraceae
		OTU1416	k__Bacteria; p__Proteobacteria; c__Alphaproteobacteria; o__Rhodobacterales; f__Rhodobacteraceae
		OTU1421	k__Bacteria; p__Proteobacteria; c__Alphaproteobacteria; o__Rhodobacterales; f__Rhodobacteraceae
		OTU1269	k__Bacteria; p__Proteobacteria; c__Alphaproteobacteria; o__Rhodobacterales; f__Rhodobacteraceae; g__*Labrenzia*
		OTU1417	k__Bacteria; p__Proteobacteria; c__Alphaproteobacteria; o__Rhodobacterales; f__Rhodobacteraceae; g__*Pseudovibrio*
		OTU1419	k__Bacteria; p__Proteobacteria; c__Alphaproteobacteria; o__Rhodobacterales; f__Rhodobacteraceae; g__*Pseudovibrio*
		OTU1422	k__Bacteria; p__Proteobacteria; c__Alphaproteobacteria; o__Rhodobacterales; f__Rhodobacteraceae; g__*Pseudovibrio*
		OTU1423	k__Bacteria; p__Proteobacteria; c__Alphaproteobacteria; o__Rhodobacterales; f__Rhodobacteraceae; g__*Pseudovibrio*

		OTU1278	k__Bacteria; p__Proteobacteria; c__Alphaproteobacteria; o__Rhodobacterales; f__Rhodobacteraceae; g__uncultured
		OTU1418	k__Bacteria; p__Proteobacteria; c__Alphaproteobacteria; o__Rhodobacterales; f__Rhodobacteraceae; g__uncultured
		OTU1233	k__Bacteria; p__Proteobacteria; c__Alphaproteobacteria; o__Sphingomonadales;f__Erythrobacteraceae; g__Altererythrobacter
		OTU1284	k__Bacteria; p__Proteobacteria; c__Gammaproteobacteria; o__Cellvibrionales; f__Halieaceae
		OTU1288	k__Bacteria; p__Proteobacteria; c__Gammaproteobacteria; o__Cellvibrionales; f__Halieaceae; g__*Parahaliea*
		OTU1276	k__Bacteria; p__Proteobacteria; c__Gammaproteobacteria; o__Cellvibrionales; f__Microbulbiferaceae; g__*Microbulbifer*
		OTU1281	k__Bacteria; p__Proteobacteria; c__Gammaproteobacteria; o__Cellvibrionales; f__Microbulbiferaceae; g__*Microbulbifer*
		OTU1283	k__Bacteria; p__Proteobacteria; c__Gammaproteobacteria; o__Cellvibrionales; f__Microbulbiferaceae; g__*Microbulbifer*
		OTU1414	k__Bacteria; p__Proteobacteria; c__Gammaproteobacteria; o__Cellvibrionales; f__Microbulbiferaceae; g__*Microbulbifer*
		OTU1420	k__Bacteria; p__Proteobacteria; c__Gammaproteobacteria; o__Cellvibrionales; f__Microbulbiferaceae; g__*Microbulbifer*
		OTU1325	k__Bacteria; p__Proteobacteria; c__Gammaproteobacteria; o__Vibrionales; f__Vibrionaceae; g__*Vibrio*

		OTU1336	k__Bacteria; p__Proteobacteria; c__Gammaproteobacteria; o__Vibrionales; f__Vibrionaceae; g__*Vibrio*
	Overlap OTUs from shallow inoculum and scraped colonies of *X. muta* community	OTU194	k__Bacteria; p__Proteobacteria; c__Gammaproteobacteria; o__Oceanospirillales; f__Halomonadaceae; g__*Halomonas*
	Overlap in OTUs derived from scraped colonies between lower and upper mesophotic of *X. muta*	OTU1240	k__Bacteria; p__Proteobacteria; c__Alphaproteobacteria; o__Rhodobacterales; f__Rhodobacteraceae; g__
		OTU1242	k__Bacteria; p__Proteobacteria; c__Alphaproteobacteria; o__Rhodobacterales; f__Rhodobacteraceae; g__
		OTU1265	k__Bacteria; p__Proteobacteria; c__Alphaproteobacteria; o__Rhodobacterales; f__Rhodobacteraceae; g__
		OTU1323	k__Bacteria; p__Proteobacteria; c__Alphaproteobacteria; o__Kordiimonadales; f__Kordiimonadaceae; g__*Kordiimonas*
		OTU1237	k__Bacteria; p__Proteobacteria; c__Gammaproteobacteria; o__Oceanospirillales; f__Hahellaceae; g__*Endozoicomonas*
		OTU1247	k__Bacteria; p__Proteobacteria; c__Gammaproteobacteria; o__Oceanospirillales; f__Hahellaceae; g__*Endozoicomonas*
		OTU1257	k__Bacteria; p__Proteobacteria; c__Gammaproteobacteria; o__Oceanospirillales; f__Hahellaceae
		OTU1358	k__Bacteria; p__Bacteroidetes; c__Flavobacteriia; o__Flavobacteriales; f__Flavobacteriaceae; g__*Aureivirga*
		OTU1238	k__Bacteria; p__Proteobacteria; c__Alphaproteobacteria; o__Rhodobacterales; f__Rhodobacteraceae

	Overlap in OTUs derived from scraped colonies between lower mesophotic and shallow *X. muta*	OTU1279	k__Bacteria; p__Proteobacteria; c__Alphaproteobacteria; o__Rhodobacterales; f__Rhodobacteraceae
		OTU1286	k__Bacteria; p__Proteobacteria; c__Alphaproteobacteria; o__Rhodobacterales; f__Rhodobacteraceae; g__uncultured
		OTU1366	k__Bacteria; p__Proteobacteria; c__Alphaproteobacteria; o__Rhodobacterales; f__Rhodobacteraceae
		OTU1277	k__Bacteria; p__Proteobacteria; c__Alphaproteobacteria; o__Sphingomonadales; f__Erythrobacteraceae; g__*Erythrobacter*
		OTU1216	k__Bacteria; p__Proteobacteria; c__Gammaproteobacteria; o__Xanthomonadales; f__Xanthomonadaceae; g__*Stenotrophomonas*
		OTU1122	k__Bacteria; p__Proteobacteria; c__Gammaproteobacteria; o__Xanthomonadales; f__Xanthomonadaceae; g__*Stenotrophomonas*
	Overlap in OTUs derived from scraped colonies between upper mesophotic and shallow *X. muta*	OTU1224	k__Bacteria; p__Actinobacteria; c__Actinobacteria; o__Micrococcales; f__Micrococcaceae; g__*Micrococcus*
		OTU1228	k__Bacteria; p__Actinobacteria; c__Actinobacteria; o__Micrococcales; f__Micrococcaceae
		OTU1200	k__Bacteria; p__Firmicutes; c__Bacilli; o__Lactobacillales; f__Lactobacillaceae; g__*Pediococcus*
		OTU1202	k__Bacteria; p__Firmicutes; c__Bacilli; o__Bacillales; f__Staphylococcaceae; g__*Staphylococcus*
		OTU1213	k__Bacteria; p__Firmicutes; c__Bacilli; o__Lactobacillales; f__Lactobacillaceae; g__*Pediococcus*

		OTU1274	k__Bacteria; p__Proteobacteria; c__Alphaproteobacteria; o__Rhodobacterales; f__Rhodobacteraceae
		OTU1285	k__Bacteria; p__Proteobacteria; c__Alphaproteobacteria; o__Rhodobacterales; f__Rhodobacteraceae
		OTU1319	k__Bacteria; p__Proteobacteria; c__Alphaproteobacteria; o__Rhodobacterales; f__Rhodobacteraceae
		OTU1326	k__Bacteria; p__Proteobacteria; c__Alphaproteobacteria; o__Rhodobacterales; f__Rhodobacteraceae; g__*Pseudovibrio*
		OTU1331	k__Bacteria; p__Proteobacteria; c__Alphaproteobacteria; o__Rhodobacterales; f__Rhodobacteraceae
		OTU1335	k__Bacteria; p__Proteobacteria; c__Alphaproteobacteria; o__Rhodobacterales; f__Rhodobacteraceae
		OTU1391	k__Bacteria; p__Proteobacteria; c__Alphaproteobacteria; o__Rhodobacterales; f__Rhodobacteraceae
		OTU1404	k__Bacteria; p__Proteobacteria; c__Alphaproteobacteria; o__Rhodobacterales; f__Rhodobacteraceae; g__*Pseudovibrio*
		OTU1268	k__Bacteria; p__Proteobacteria; c__Gammaproteobacteria; o__Cellvibrionales; f__Microbulbiferaceae; g__*Microbulbifer*
		OTU1270	k__Bacteria; p__Proteobacteria; c__Gammaproteobacteria; o__Cellvibrionales; f__Microbulbiferaceae; g__*Microbulbifer*
		OTU1271	k__Bacteria; p__Proteobacteria; c__Gammaproteobacteria; o__Cellvibrionales; f__Microbulbiferaceae; g__*Microbulbifer*

		OTU1275	k__Bacteria; p__Proteobacteria; c__Gammaproteobacteria; o__Cellvibrionales; f__Microbulbiferaceae; g__*Microbulbifer*
		OTU1318	k__Bacteria; p__Proteobacteria; c__Gammaproteobacteria; o__Cellvibrionales; f__Microbulbiferaceae; g__*Microbulbifer*
		OTU1320	k__Bacteria; p__Proteobacteria; c__Gammaproteobacteria; o__Cellvibrionales; f__Microbulbiferaceae; g__*Microbulbifer*
		OTU1321	k__Bacteria; p__Proteobacteria; c__Gammaproteobacteria; o__Cellvibrionales; f__Microbulbiferaceae; g__*Microbulbifer*
		OTU1324	k__Bacteria; p__Proteobacteria; c__Gammaproteobacteria; o__Cellvibrionales; f__Microbulbiferaceae; g__*Microbulbifer*
		OTU1332	k__Bacteria; p__Proteobacteria; c__Gammaproteobacteria; o__Cellvibrionales; f__Microbulbiferaceae; g__*Microbulbifer*
	Overlap in OTUs derived from scraped colonies among lower mesophotic, upper mesophotic and shallow *X. muta*	OTU1201	k__Bacteria; p__Firmicutes; c__Bacilli; o__Bacillales; f__Bacillaceae; g__*Aeribacillus*
		OTU1223	k__Bacteria; p__Proteobacteria; c__Alphaproteobacteria; o__Sphingomonadales; f__Erythrobacteraceae; *g__Erythrobacter*
		OTU512	k__Bacteria; p__Proteobacteria; c__Alphaproteobacteria; o__Rhodobacterales; f__Rhodobacteraceae
		OTU592	k__Bacteria; p__Proteobacteria; c__Alphaproteobacteria; o__Rhodobacterales; f__Rhodobacteraceae; g__*Ruegeria*
		OTU1234	k__Bacteria; p__Proteobacteria; c__Alphaproteobacteria; o__Rhodobacterales; f__Rhodobacteraceae; g__*Pseudovibrio*

		OTU1239	k__Bacteria; p__Proteobacteria; c__Alphaproteobacteria; o__Rhodobacterales; f__Rhodobacteraceae; g__*Pseudovibrio*
		OTU1241	k__Bacteria; p__Proteobacteria; c__Alphaproteobacteria; o__Rhodobacterales; f__Rhodobacteraceae; g__*Pseudovibrio*
		OTU1245	k__Bacteria; p__Proteobacteria; c__Alphaproteobacteria; o__Rhodobacterales; f__Rhodobacteraceae
		OTU1249	k__Bacteria; p__Proteobacteria; c__Alphaproteobacteria; o__Rhodobacterales; f__Rhodobacteraceae; g__*Pseudovibrio*
		OTU1255	k__Bacteria; p__Proteobacteria; c__Alphaproteobacteria; o__Rhodobacterales; f__Rhodobacteraceae; g__*Pseudovibrio*
		OTU1272	k__Bacteria; p__Proteobacteria; c__Alphaproteobacteria; o__Rhodobacterales; f__Rhodobacteraceae; g__uncultured
		OTU1273	k__Bacteria; p__Proteobacteria; c__Alphaproteobacteria; o__Rhodobacterales; f__Rhodobacteraceae
		OTU194	k__Bacteria; p__Proteobacteria; c__Gammaproteobacteria; o__Oceanospirillales; f__Halomonadaceae; g__*Halomonas*
A. sventres	OTUs present in scraped colonies of upper mesophotic *A.sventres*	OTU1804	k__Bacteria; p__Actinobacteria; c__Actinobacteria; o__Corynebacteriales
		OTU1653	k__Bacteria; p__Actinobacteria; c__Actinobacteria; o__Corynebacteriales; f__*Nocardia*ceae; g__*Nocardia*
		OTU1523	k__Bacteria; p__Actinobacteria; c__Actinobacteria; o__Corynebacteriales; f__*Nocardia*ceae; g__*Rhodococcus*
		OTU1799	k__Bacteria; p__Actinobacteria; c__Actinobacteria; o__Micrococcales;

		OTU1802	k__Bacteria; p__Actinobacteria; c__Actinobacteria; o__Micrococcales;
		OTU1803	k__Bacteria; p__Actinobacteria; c__Actinobacteria; o__Micrococcales;
		OTU1228	k__Bacteria; p__Actinobacteria; c__Actinobacteria; o__Micrococcales; f__Micrococcaceae
		OTU1344	k__Bacteria; p__Actinobacteria; c__Actinobacteria; o__Micrococcales; f__Micrococcaceae
		OTU1350	k__Bacteria; p__Actinobacteria; c__Actinobacteria; o__Micrococcales; f__Micrococcaceae
		OTU1808	k__Bacteria; p__Actinobacteria; c__Actinobacteria; o__Micrococcales; f__Micrococcaceae
		OTU1224	k__Bacteria; p__Actinobacteria; c__Actinobacteria; o__Micrococcales; f__Micrococcaceae; g__*Micrococcus*
		OTU1346	k__Bacteria; p__Actinobacteria; c__Actinobacteria; o__Micrococcales; f__Micrococcaceae; g__*Micrococcus*
		OTU1349	k__Bacteria; p__Actinobacteria; c__Actinobacteria; o__Micrococcales; f__Micrococcaceae; g__*Micrococcus*
		OTU1352	k__Bacteria; p__Actinobacteria; c__Actinobacteria; o__Micrococcales; f__Micrococcaceae; g__*Micrococcus*
		OTU1353	k__Bacteria; p__Actinobacteria; c__Actinobacteria; o__Micrococcales; f__Micrococcaceae; g__*Micrococcus*
		OTU1800	k__Bacteria; p__Actinobacteria; c__Actinobacteria; o__Micrococcales; f__Micrococcaceae; g__*Micrococcus*

		OTU1358	k__Bacteria; p__Bacteroidetes; c__Flavobacteriia; o__Flavobacteriales; f__Flavobacteriaceae; g__*Aureivirga*
		OTU1654	k__Bacteria; p__Bacteroidetes; c__Flavobacteriia; o__Flavobacteriales; f__Flavobacteriaceae; g__*Aureivirga*
		OTU1656	k__Bacteria; p__Bacteroidetes; c__Flavobacteriia; o__Flavobacteriales; f__Flavobacteriaceae; g__*Aureivirga*
		OTU1532	k__Bacteria; p__Bacteroidetes; c__Flavobacteriia; o__Flavobacteriales; f__Flavobacteriaceae; g__*Muricauda*
		OTU1535	k__Bacteria; p__Bacteroidetes; c__Flavobacteriia; o__Flavobacteriales; f__Flavobacteriaceae; g__uncultured_bacterium
		OTU1074	k__Bacteria; p__Cyanobacteria; c__Cyanobacteria; o__SubsectionI; f__FamilyI; g__*Synechococcus*
		OTU1805	k__Bacteria; p__Firmicutes; c__Bacilli; o__Bacillales; f__Staphylococcaceae
		OTU1202	k__Bacteria; p__Firmicutes; c__Bacilli; o__Bacillales; f__Staphylococcaceae; g__*Staphylococcus*
		OTU1347	k__Bacteria; p__Firmicutes; c__Bacilli; o__Bacillales; f__Staphylococcaceae; g__*Staphylococcus*
		OTU1797	k__Bacteria; p__Firmicutes; c__Bacilli; o__Bacillales; f__Staphylococcaceae; g__*Staphylococcus*
		OTU1798	k__Bacteria; p__Firmicutes; c__Bacilli; o__Bacillales; f__Staphylococcaceae; g__*Staphylococcus*

		OTU1801	k__Bacteria; p__Firmicutes; c__Bacilli; o__Bacillales; f__Staphylococcaceae; g__*Staphylococcus*
		OTU1806	k__Bacteria; p__Firmicutes; c__Bacilli; o__Bacillales; f__Staphylococcaceae; g__*Staphylococcus*
		OTU1807	k__Bacteria; p__Firmicutes; c__Bacilli; o__Bacillales; f__Staphylococcaceae; g__*Staphylococcus*
		OTU1213	k__Bacteria; p__Firmicutes; c__Bacilli; o__Lactobacillales; f__Lactobacillaceae; g__*Pediococcus*
		OTU1525	k__Bacteria; p__Proteobacteria; c__Alphaproteobacteria; o__Rhizobiales; f__OCS116_clade; g__uncultured_bacterium
		OTU1238	k__Bacteria; p__Proteobacteria; c__Alphaproteobacteria; o__Rhodobacterales; f__Rhodobacteraceae
		OTU1273	k__Bacteria; p__Proteobacteria; c__Alphaproteobacteria; o__Rhodobacterales; f__Rhodobacteraceae
		OTU1280	k__Bacteria; p__Proteobacteria; c__Alphaproteobacteria; o__Rhodobacterales; f__Rhodobacteraceae
		OTU1335	k__Bacteria; p__Proteobacteria; c__Alphaproteobacteria; o__Rhodobacterales; f__Rhodobacteraceae
		OTU1391	k__Bacteria; p__Proteobacteria; c__Alphaproteobacteria; o__Rhodobacterales; f__Rhodobacteraceae
		OTU1482	k__Bacteria; p__Proteobacteria; c__Alphaproteobacteria; o__Rhodobacterales; f__Rhodobacteraceae

		OTU1594	k__Bacteria; p__Proteobacteria; c__Alphaproteobacteria; o__Rhodobacterales; f__Rhodobacteraceae
		OTU1655	k__Bacteria; p__Proteobacteria; c__Alphaproteobacteria; o__Rhodobacterales; f__Rhodobacteraceae
		OTU1579	k__Bacteria; p__Proteobacteria; c__Alphaproteobacteria; o__Rhodobacterales; f__Rhodobacteraceae; g__*Paracoccus*
		OTU1595	k__Bacteria; p__Proteobacteria; c__Alphaproteobacteria; o__Rhodobacterales; f__Rhodobacteraceae; g__*Paracoccus*
		OTU1772	k__Bacteria; p__Proteobacteria; c__Alphaproteobacteria; o__Rhodobacterales; f__Rhodobacteraceae; g__*Paracoccus*
		OTU1774	k__Bacteria; p__Proteobacteria; c__Alphaproteobacteria; o__Rhodobacterales; f__Rhodobacteraceae; g__*Paracoccus*
		OTU1776	k__Bacteria; p__Proteobacteria; c__Alphaproteobacteria; o__Rhodobacterales; f__Rhodobacteraceae; g__*Paracoccus*
		OTU1526	k__Bacteria; p__Proteobacteria; c__Alphaproteobacteria; o__Rhodobacterales; f__Rhodobacteraceae; g__*Pseudovibrio*
		OTU1533	k__Bacteria; p__Proteobacteria; c__Alphaproteobacteria; o__Rhodobacterales; f__Rhodobacteraceae; g__*Pseudovibrio*
		OTU1591	k__Bacteria; p__Proteobacteria; c__Alphaproteobacteria; o__Rhodobacterales; f__Rhodobacteraceae; g__*Pseudovibrio*
		OTU1534	k__Bacteria; p__Proteobacteria; c__Alphaproteobacteria; o__Rhodobacterales; f__Rhodobacteraceae; g__uncultured

		OTU1771	k__Bacteria; p__Proteobacteria; c__Alphaproteobacteria; o__Sphingomonadales; f__Sphingomonadaceae; g__*Sphingomonas*
		OTU1773	k__Bacteria; p__Proteobacteria; c__Alphaproteobacteria; o__Sphingomonadales; f__Sphingomonadaceae; g__*Sphingomonas*
		OTU1775	k__Bacteria; p__Proteobacteria; c__Alphaproteobacteria; o__Sphingomonadales; f__Sphingomonadaceae; g__*Sphingomonas*
		OTU1777	k__Bacteria; p__Proteobacteria; c__Alphaproteobacteria; o__Sphingomonadales; f__Sphingomonadaceae; g__*Sphingomonas*
		OTU1275	k__Bacteria; p__Proteobacteria; c__Gammaproteobacteria; o__Cellvibrionales; f__Microbulbiferaceae; g__*Microbulbifer*
		OTU1283	k__Bacteria; p__Proteobacteria; c__Gammaproteobacteria; o__Cellvibrionales; f__Microbulbiferaceae; g__*Microbulbifer*
		OTU1420	k__Bacteria; p__Proteobacteria; c__Gammaproteobacteria; o__Cellvibrionales; f__Microbulbiferaceae; g__*Microbulbifer*
		OTU1528	k__Bacteria; p__Proteobacteria; c__Gammaproteobacteria; o__Cellvibrionales; f__Microbulbiferaceae; g__*Microbulbifer*
		OTU1531	k__Bacteria; p__Proteobacteria; c__Gammaproteobacteria; o__Cellvibrionales; f__Microbulbiferaceae; g__*Microbulbifer*
		OTU1236	k__Bacteria; p__Proteobacteria; c__Gammaproteobacteria; o__Oceanospirillales; f__Alcanivoracaceae; g__*Alcanivorax*
		OTU620	k__Bacteria; p__Proteobacteria; c__Gammaproteobacteria; o__Oceanospirillales; f__Hahellaceae; g__*Endozoicomonas*

	OTUs present in scraped colonies of shallow *A.sventres*	OTU1668	k__Bacteria; p__Actinobacteria; c__Actinobacteria; o__Micrococcales; f__Intrasporangiaceae
		OTU1666	k__Bacteria; p__Actinobacteria; c__Actinobacteria; o__Micrococcales; f__Intrasporangiaceae; g__*Janibacter*
		OTU1667	k__Bacteria; p__Actinobacteria; c__Actinobacteria; o__Micrococcales; f__Intrasporangiaceae; g__*Janibacter*
		OTU1670	k__Bacteria; p__Actinobacteria; c__Actinobacteria; o__Micrococcales; f__Intrasporangiaceae; g__*Janibacter*
		OTU1788	k__Bacteria; p__Actinobacteria; c__Actinobacteria; o__Micromonosporales;f__Micromonosporaceae; g__*Micromonospora*
		OTU1555	k__Bacteria; p__Bacteroidetes; c__Cytophagia; o__Cytophagales; f__Flammeovirgaceae; g__*Fulvitalea*
		OTU1549	k__Bacteria; p__Bacteroidetes; c__Flavobacteriia; o__Flavobacteriales; f__Flavobacteriaceae; g__*Aureivirga*
		OTU1665	k__Bacteria; p__Bacteroidetes; c__Flavobacteriia; o__Flavobacteriales; f__Flavobacteriaceae; g__*Aureivirga*
		OTU1669	k__Bacteria; p__Bacteroidetes; c__Flavobacteriia; o__Flavobacteriales; f__Flavobacteriaceae; g__*Aureivirga*
		OTU1795	k__Bacteria; p__Firmicutes; c__Bacilli
		OTU1792	k__Bacteria; p__Firmicutes; c__Bacilli; o__Lactobacillales; f__Lactobacillaceae
		OTU1200	k__Bacteria; p__Firmicutes; c__Bacilli; o__Lactobacillales; f__Lactobacillaceae; g__*Pediococcus*

		OTU1796	k__Bacteria; p__Firmicutes; c__Bacilli; o__Lactobacillales; f__Lactobacillaceae; g__*Pediococcus*
		OTU1390	k__Bacteria; p__Proteobacteria; c__Alphaproteobacteria
		OTU1572	k__Bacteria; p__Proteobacteria; c__Alphaproteobacteria
		OTU1573	k__Bacteria; p__Proteobacteria; c__Alphaproteobacteria
		OTU1599	k__Bacteria; p__Proteobacteria; c__Alphaproteobacteria; o__Rhizobiales; f__Brucellaceae
		OTU1780	k__Bacteria; p__Proteobacteria; c__Alphaproteobacteria; o__Rhizobiales; f__Brucellaceae
		OTU1781	k__Bacteria; p__Proteobacteria; c__Alphaproteobacteria; o__Rhizobiales; f__Brucellaceae
		OTU1784	k__Bacteria; p__Proteobacteria; c__Alphaproteobacteria; o__Rhizobiales; f__Brucellaceae
		OTU1786	k__Bacteria; p__Proteobacteria; c__Alphaproteobacteria; o__Rhizobiales; f__Brucellaceae
		OTU1790	k__Bacteria; p__Proteobacteria; c__Alphaproteobacteria; o__Rhizobiales; f__Brucellaceae
		OTU1566	k__Bacteria; p__Proteobacteria; c__Alphaproteobacteria; o__Rhizobiales; f__Phyllobacteriaceae; g__ *Mesorhizobium*
		OTU1598	k__Bacteria; p__Proteobacteria; c__Alphaproteobacteria; o__Rhizobiales; f__Phyllobacteriaceae; g__*Phyllobacterium*
		OTU1604	k__Bacteria; p__Proteobacteria; c__Alphaproteobacteria; o__Rhizobiales; f__Rhizobiaceae

		OTU1608	k__Bacteria; p__Proteobacteria; c__Alphaproteobacteria; o__Rhizobiales; f__Rhizobiaceae
		OTU1779	k__Bacteria; p__Proteobacteria; c__Alphaproteobacteria; o__Rhizobiales; f__Rhizobiaceae
		OTU1785	k__Bacteria; p__Proteobacteria; c__Alphaproteobacteria; o__Rhizobiales; f__Rhizobiaceae
		OTU1597	k__Bacteria; p__Proteobacteria; c__Alphaproteobacteria; o__Rhizobiales; f__Rhizobiaceae; g__*Rhizobium*
		OTU930	k__Bacteria; p__Proteobacteria; c__Alphaproteobacteria; o__Rhodobacterales; f__Rhodobacteraceae
		OTU1240	k__Bacteria; p__Proteobacteria; c__Alphaproteobacteria; o__Rhodobacterales; f__Rhodobacteraceae
		OTU1426	k__Bacteria; p__Proteobacteria; c__Alphaproteobacteria; o__Rhodobacterales; f__Rhodobacteraceae
		OTU1543	k__Bacteria; p__Proteobacteria; c__Alphaproteobacteria; o__Rhodobacterales; f__Rhodobacteraceae
		OTU1553	k__Bacteria; p__Proteobacteria; c__Alphaproteobacteria; o__Rhodobacterales; f__Rhodobacteraceae
		OTU1605	k__Bacteria; p__Proteobacteria; c__Alphaproteobacteria; o__Rhodobacterales; f__Rhodobacteraceae
		OTU1606	k__Bacteria; p__Proteobacteria; c__Alphaproteobacteria; o__Rhodobacterales; f__Rhodobacteraceae

		OTU1607	k__Bacteria; p__Proteobacteria; c__Alphaproteobacteria; o__Rhodobacterales; f__Rhodobacteraceae
		OTU1405	k__Bacteria; p__Proteobacteria; c__Alphaproteobacteria; o__Rhodobacterales; f__Rhodobacteraceae; g__*Paracoccus*
		OTU1550	k__Bacteria; p__Proteobacteria; c__Alphaproteobacteria; o__Rhodobacterales; f__Rhodobacteraceae; g__*Pseudovibrio*
		OTU1570	k__Bacteria; p__Proteobacteria; c__Alphaproteobacteria; o__Rhodobacterales; f__Rhodobacteraceae; g__*Pseudovibrio*
		OTU1571	k__Bacteria; p__Proteobacteria; c__Alphaproteobacteria; o__Rhodobacterales; f__Rhodobacteraceae; g__*Pseudovibrio*
		OTU1600	k__Bacteria; p__Proteobacteria; c__Alphaproteobacteria; o__Rhodobacterales; f__Rhodobacteraceae; g__*Pseudovibrio*
		OTU1601	k__Bacteria; p__Proteobacteria; c__Alphaproteobacteria; o__Rhodobacterales; f__Rhodobacteraceae; g__*Pseudovibrio*
		OTU1602	k__Bacteria; p__Proteobacteria; c__Alphaproteobacteria; o__Rhodobacterales; f__Rhodobacteraceae; g__*Pseudovibrio*
		OTU1610	k__Bacteria; p__Proteobacteria; c__Alphaproteobacteria; o__Rhodobacterales; f__Rhodobacteraceae; g__*Pseudovibrio*
		OTU1658	k__Bacteria; p__Proteobacteria; c__Alphaproteobacteria; o__Rhodobacterales; f__Rhodobacteraceae; g__*Pseudovibrio*
		OTU1659	k__Bacteria; p__Proteobacteria; c__Alphaproteobacteria; o__Rhodobacterales; f__Rhodobacteraceae; g__*Pseudovibrio*

		OTU1660	k__Bacteria; p__Proteobacteria; c__Alphaproteobacteria; o__Rhodobacterales; f__Rhodobacteraceae; g__*Pseudovibrio*
		OTU1664	k__Bacteria; p__Proteobacteria; c__Alphaproteobacteria; o__Rhodobacterales; f__Rhodobacteraceae; g__*Pseudovibrio*
		OTU1223	k__Bacteria; p__Proteobacteria; c__Alphaproteobacteria; o__Sphingomonadales; f__Erythrobacteraceae; g__*Erythrobacter*
		OTU1568	k__Bacteria; p__Proteobacteria; c__Alphaproteobacteria; o__Sphingomonadales; f__Erythrobacteraceae; g__*Erythrobacter*
		OTU1569	k__Bacteria; p__Proteobacteria; c__Alphaproteobacteria; o__Sphingomonadales; f__Erythrobacteraceae; g__*Erythrobacter*
		OTU1546	k__Bacteria; p__Proteobacteria; c__Gammaproteobacteria
		OTU1657	k__Bacteria; p__Proteobacteria; c__Gammaproteobacteria
		OTU1281	k__Bacteria; p__Proteobacteria; c__Gammaproteobacteria; o__Cellvibrionales; f__Microbulbiferaceae; g__*Microbulbifer*
		OTU1403	k__Bacteria; p__Proteobacteria; c__Gammaproteobacteria; o__Cellvibrionales; f__Microbulbiferaceae; g__*Microbulbifer*
		OTU1431	k__Bacteria; p__Proteobacteria; c__Gammaproteobacteria; o__Cellvibrionales; f__Microbulbiferaceae; g__*Microbulbifer*
		OTU1541	k__Bacteria; p__Proteobacteria; c__Gammaproteobacteria; o__Cellvibrionales; f__Microbulbiferaceae; g__*Microbulbifer*
		OTU1551	k__Bacteria; p__Proteobacteria; c__Gammaproteobacteria; o__Cellvibrionales; f__Microbulbiferaceae; g__*Microbulbifer*

		OTU1556	k__Bacteria; p__Proteobacteria; c__Gammaproteobacteria; o__Cellvibrionales; f__Microbulbiferaceae; g__*Microbulbifer*
		OTU1603	k__Bacteria; p__Proteobacteria; c__Gammaproteobacteria; o__Cellvibrionales; f__Microbulbiferaceae; g__*Microbulbifer*
		OTU1661	k__Bacteria; p__Proteobacteria; c__Gammaproteobacteria; o__Cellvibrionales; f__Microbulbiferaceae; g__*Microbulbifer*
		OTU1662	k__Bacteria; p__Proteobacteria; c__Gammaproteobacteria; o__Cellvibrionales; f__Microbulbiferaceae; g__*Microbulbifer*
		OTU1536	k__Bacteria; p__Proteobacteria; c__Gammaproteobacteria; o__Enterobacteriales; f__Enterobacteriaceae
		OTU1537	k__Bacteria; p__Proteobacteria; c__Gammaproteobacteria; o__Enterobacteriales; f__Enterobacteriaceae
		OTU1538	k__Bacteria; p__Proteobacteria; c__Gammaproteobacteria; o__Enterobacteriales; f__Enterobacteriaceae
		OTU1539	k__Bacteria; p__Proteobacteria; c__Gammaproteobacteria; o__Enterobacteriales; f__Enterobacteriaceae
		OTU1540	k__Bacteria; p__Proteobacteria; c__Gammaproteobacteria; o__Enterobacteriales; f__Enterobacteriaceae
		OTU1542	k__Bacteria; p__Proteobacteria; c__Gammaproteobacteria; o__Enterobacteriales; f__Enterobacteriaceae
		OTU1544	k__Bacteria; p__Proteobacteria; c__Gammaproteobacteria; o__Enterobacteriales; f__Enterobacteriaceae

		OTU1545	k__Bacteria; p__Proteobacteria; c__Gammaproteobacteria; o__Enterobacteriales; f__Enterobacteriaceae
		OTU1778	k__Bacteria; p__Proteobacteria; c__Gammaproteobacteria; o__Enterobacteriales; f__Enterobacteriaceae
		OTU1782	k__Bacteria; p__Proteobacteria; c__Gammaproteobacteria; o__Enterobacteriales; f__Enterobacteriaceae
		OTU1783	k__Bacteria; p__Proteobacteria; c__Gammaproteobacteria; o__Enterobacteriales; f__Enterobacteriaceae
		OTU1787	k__Bacteria; p__Proteobacteria; c__Gammaproteobacteria; o__Enterobacteriales; f__Enterobacteriaceae
		OTU1789	k__Bacteria; p__Proteobacteria; c__Gammaproteobacteria; o__Enterobacteriales; f__Enterobacteriaceae
		OTU1791	k__Bacteria; p__Proteobacteria; c__Gammaproteobacteria; o__Enterobacteriales; f__Enterobacteriaceae; g__*Escherichia-Shigella*
		OTU1794	k__Bacteria; p__Proteobacteria; c__Gammaproteobacteria; o__Enterobacteriales; f__Enterobacteriaceae; g__*Escherichia-Shigella*
		OTU1554	k__Bacteria; p__Proteobacteria; c__Gammaproteobacteria; o__Vibrionales; f__Vibrionaceae
		OTU1564	k__Bacteria; p__Proteobacteria; c__Gammaproteobacteria; o__Vibrionales; f__Vibrionaceae
		OTU1565	k__Bacteria; p__Proteobacteria; c__Gammaproteobacteria; o__Vibrionales; f__Vibrionaceae

		OTU1793	k__Bacteria; p__Proteobacteria; c__Gammaproteobacteria; o__Vibrionales; f__Vibrionaceae
		OTU820	k__Bacteria; p__Proteobacteria; c__Gammaproteobacteria; o__Vibrionales; f__Vibrionaceae; g__*Vibrio*
		OTU1548	k__Bacteria; p__Proteobacteria; c__Gammaproteobacteria; o__Vibrionales; f__Vibrionaceae; g__*Vibrio*
		OTU1552	k__Bacteria; p__Proteobacteria; c__Gammaproteobacteria; o__Vibrionales; f__Vibrionaceae; g__*Vibrio*
		OTU1558	k__Bacteria; p__Proteobacteria; c__Gammaproteobacteria; o__Vibrionales; f__Vibrionaceae; g__*Vibrio*
		OTU1559	k__Bacteria; p__Proteobacteria; c__Gammaproteobacteria; o__Vibrionales; f__Vibrionaceae; g__*Vibrio*
		OTU1560	k__Bacteria; p__Proteobacteria; c__Gammaproteobacteria; o__Vibrionales; f__Vibrionaceae; g__*Vibrio*
		OTU1561	k__Bacteria; p__Proteobacteria; c__Gammaproteobacteria; o__Vibrionales; f__Vibrionaceae; g__*Vibrio*
		OTU1562	k__Bacteria; p__Proteobacteria; c__Gammaproteobacteria; o__Vibrionales; f__Vibrionaceae; g__*Vibrio*
		OTU1563	k__Bacteria; p__Proteobacteria; c__Gammaproteobacteria; o__Vibrionales; f__Vibrionaceae; g__*Vibrio*
		OTU1567	k__Bacteria; p__Proteobacteria; c__Gammaproteobacteria; o__Vibrionales; f__Vibrionaceae; g__*Vibrio*

		OTU1574	k__Bacteria; p__Proteobacteria; c__Gammaproteobacteria; o__Vibrionales; f__Vibrionaceae; g__*Vibrio*
		OTU1609	k__Bacteria; p__Proteobacteria; c__Gammaproteobacteria; o__Vibrionales; f__Vibrionaceae; g__*Vibrio*
		OTU1611	k__Bacteria; p__Proteobacteria; c__Gammaproteobacteria; o__Vibrionales; f__Vibrionaceae; g__*Vibrio*
		OTU1557	NA; p__; c__; o__; f__; g__
		OTU1671	NA; p__; c__; o__; f__; g__
	Overlap in OTUs fromupper mesophotic *A. sventres* between inoculum and scraped plates	OTU1404	k__Bacteria; p__Proteobacteria; c__Alphaproteobacteria; o__Rhodobacterales; f__Rhodobacteraceae; g__*Pseudovibrio*
		OTU514	k__Bacteria; p__Proteobacteria; c__Gammaproteobacteria; o__Oceanospirillales; f__Hahellaceae; g__*Endozoicomonas*
		OTU620	k__Bacteria; p__Proteobacteria; c__Gammaproteobacteria; o__Oceanospirillales; f__Hahellaceae; g__*Endozoicomonas*
	Overlap in OTUs from shallow *A. sventres* between inoculum and scraped plates	OTU514	k__Bacteria; p__Proteobacteria; c__Gammaproteobacteria; o__Oceanospirillales; f__Hahellaceae; g__*Endozoicomonas*
		OTU816	k__Bacteria; p__Cyanobacteria; c__Cyanobacteria; o__SubsectionI; f__FamilyI; g__*Synechococcus*
	Overlap in OTUs derived from scraped plates between upper mesophotic and shallow *A. sventres*	OTU1663	k__Bacteria; p__Actinobacteria; c__Actinobacteria; o__Micrococcales; f__Intrasporangiaceae; g__*Janibacter*
		OTU816	k__Bacteria; p__Cyanobacteria; c__Cyanobacteria; o__SubsectionI; f__FamilyI; g__*Synechococcus*

		OTU512	k__Bacteria; p__Proteobacteria; c__Alphaproteobacteria; o__Rhodobacterales; f__Rhodobacteraceae
		OTU1245	k__Bacteria; p__Proteobacteria; c__Alphaproteobacteria; o__Rhodobacterales; f__Rhodobacteraceae
		OTU1265	k__Bacteria; p__Proteobacteria; c__Alphaproteobacteria; o__Rhodobacterales; f__Rhodobacteraceae
		OTU1285	k__Bacteria; p__Proteobacteria; c__Alphaproteobacteria; o__Rhodobacterales; f__Rhodobacteraceae
		OTU1319	k__Bacteria; p__Proteobacteria; c__Alphaproteobacteria; o__Rhodobacterales; f__Rhodobacteraceae
		OTU1331	k__Bacteria; p__Proteobacteria; c__Alphaproteobacteria; o__Rhodobacterales; f__Rhodobacteraceae
		OTU1366	k__Bacteria; p__Proteobacteria; c__Alphaproteobacteria; o__Rhodobacterales; f__Rhodobacteraceae
		OTU1392	k__Bacteria; p__Proteobacteria; c__Alphaproteobacteria; o__Rhodobacterales; f__Rhodobacteraceae
		OTU1397	k__Bacteria; p__Proteobacteria; c__Alphaproteobacteria; o__Rhodobacterales; f__Rhodobacteraceae
		OTU1547	k__Bacteria; p__Proteobacteria; c__Alphaproteobacteria; o__Rhodobacterales; f__Rhodobacteraceae
		OTU1592	k__Bacteria; p__Proteobacteria; c__Alphaproteobacteria; o__Rhodobacterales; f__Rhodobacteraceae

		OTU1593	k__Bacteria; p__Proteobacteria; c__Alphaproteobacteria; o__Rhodobacterales; f__Rhodobacteraceae
		OTU1317	k__Bacteria; p__Proteobacteria; c__Alphaproteobacteria; o__Rhodobacterales; f__Rhodobacteraceae; g__*Labrenzia*
		OTU1234	k__Bacteria; p__Proteobacteria; c__Alphaproteobacteria; o__Rhodobacterales; f__Rhodobacteraceae; g__*Pseudovibrio*
		OTU1239	k__Bacteria; p__Proteobacteria; c__Alphaproteobacteria; o__Rhodobacterales; f__Rhodobacteraceae; g__*Pseudovibrio*
		OTU1241	k__Bacteria; p__Proteobacteria; c__Alphaproteobacteria; o__Rhodobacterales; f__Rhodobacteraceae; g__*Pseudovibrio*
		OTU1249	k__Bacteria; p__Proteobacteria; c__Alphaproteobacteria; o__Rhodobacterales; f__Rhodobacteraceae; g__*Pseudovibrio*
		OTU1255	k__Bacteria; p__Proteobacteria; c__Alphaproteobacteria; o__Rhodobacterales; f__Rhodobacteraceae; g__*Pseudovibrio*
		OTU1326	k__Bacteria; p__Proteobacteria; c__Alphaproteobacteria; o__Rhodobacterales; f__Rhodobacteraceae; g__*Pseudovibrio*
		OTU1389	k__Bacteria; p__Proteobacteria; c__Alphaproteobacteria; o__Rhodobacterales; f__Rhodobacteraceae; g__*Pseudovibrio*
		OTU1404	k__Bacteria; p__Proteobacteria; c__Alphaproteobacteria; o__Rhodobacterales; f__Rhodobacteraceae; g__*Pseudovibrio*
		OTU1413	k__Bacteria; p__Proteobacteria; c__Alphaproteobacteria; o__Rhodobacterales; f__Rhodobacteraceae; g__*Pseudovibrio*

		OTU1427	k__Bacteria; p__Proteobacteria; c__Alphaproteobacteria; o__Rhodobacterales; f__Rhodobacteraceae; g__*Pseudovibrio*
		OTU1429	k__Bacteria; p__Proteobacteria; c__Alphaproteobacteria; o__Rhodobacterales; f__Rhodobacteraceae; g__*Pseudovibrio*
		OTU1522	k__Bacteria; p__Proteobacteria; c__Alphaproteobacteria; o__Rhodobacterales; f__Rhodobacteraceae; g__*Pseudovibrio*
		OTU1524	k__Bacteria; p__Proteobacteria; c__Alphaproteobacteria; o__Rhodobacterales; f__Rhodobacteraceae; g__*Pseudovibrio*
		OTU1527	k__Bacteria; p__Proteobacteria; c__Alphaproteobacteria; o__Rhodobacterales; f__Rhodobacteraceae; g__*Pseudovibrio*
		OTU1596	k__Bacteria; p__Proteobacteria; c__Alphaproteobacteria; o__Rhodobacterales; f__Rhodobacteraceae; g__*Pseudovibrio*
		OTU592	k__Bacteria; p__Proteobacteria; c__Alphaproteobacteria; o__Rhodobacterales; f__Rhodobacteraceae; g__*Ruegeria*
		OTU1272	k__Bacteria; p__Proteobacteria; c__Alphaproteobacteria; o__Rhodobacterales; f__Rhodobacteraceae; g__uncultured
		OTU1632	k__Bacteria; p__Proteobacteria; c__Betaproteobacteria; o__Burkholderiales; f__Burkholderiaceae; g__*Limnobacter*
		OTU1268	k__Bacteria; p__Proteobacteria; c__Gammaproteobacteria; o__Cellvibrionales; f__Microbulbiferaceae; g__*Microbulbifer*
		OTU1270	k__Bacteria; p__Proteobacteria; c__Gammaproteobacteria; o__Cellvibrionales; f__Microbulbiferaceae; g__*Microbulbifer*

		OTU1271	k__Bacteria; p__Proteobacteria; c__Gammaproteobacteria; o__Cellvibrionales; f__Microbulbiferaceae; g__*Microbulbifer*
		OTU1276	k__Bacteria; p__Proteobacteria; c__Gammaproteobacteria; o__Cellvibrionales; f__Microbulbiferaceae; g__*Microbulbifer*
		OTU1318	k__Bacteria; p__Proteobacteria; c__Gammaproteobacteria; o__Cellvibrionales; f__Microbulbiferaceae; g__*Microbulbifer*
		OTU1320	k__Bacteria; p__Proteobacteria; c__Gammaproteobacteria; o__Cellvibrionales; f__Microbulbiferaceae; g__*Microbulbifer*
		OTU1321	k__Bacteria; p__Proteobacteria; c__Gammaproteobacteria; o__Cellvibrionales; f__Microbulbiferaceae; g__*Microbulbifer*
		OTU1324	k__Bacteria; p__Proteobacteria; c__Gammaproteobacteria; o__Cellvibrionales; f__Microbulbiferaceae; g__*Microbulbifer*
		OTU1332	k__Bacteria; p__Proteobacteria; c__Gammaproteobacteria; o__Cellvibrionales; f__Microbulbiferaceae; g__*Microbulbifer*
		OTU1407	k__Bacteria; p__Proteobacteria; c__Gammaproteobacteria; o__Cellvibrionales; f__Microbulbiferaceae; g__*Microbulbifer*
		OTU1411	k__Bacteria; p__Proteobacteria; c__Gammaproteobacteria; o__Cellvibrionales; f__Microbulbiferaceae; g__*Microbulbifer*
		OTU1414	k__Bacteria; p__Proteobacteria; c__Gammaproteobacteria; o__Cellvibrionales; f__Microbulbiferaceae; g__*Microbulbifer*
		OTU1430	k__Bacteria; p__Proteobacteria; c__Gammaproteobacteria; o__Cellvibrionales; f__Microbulbiferaceae; g__*Microbulbifer*

		OTU1529	k__Bacteria; p__Proteobacteria; c__Gammaproteobacteria; o__Cellvibrionales; f__Microbulbiferaceae; g__*Microbulbifer*
		OTU514	k__Bacteria; p__Proteobacteria; c__Gammaproteobacteria; o__Oceanospirillales; f__Hahellaceae; g__*Endozoicomonas*
		OTU1530	k__Bacteria; p__Proteobacteria; c__Gammaproteobacteria; o__Oceanospirillales; f__Hahellaceae; g__*Endozoicomonas*

marine drugs

MDPI

Table S3. Number of colonies counted from each sponge per agar medium from agar plates dedicated for picking. Panel A shows colonies from *X.muta* and Panel B indicates colonies from *A. sventres*.

A. *X. muta*

Picked isolates	*X. muta* lower mesophotic			*X. muta* upper mesophotic			*X. muta* shallow			Total per medium
	XM3	XM4	XM5	XM7	XM8	XM9	XM12	XM14	XM15	
MA1/10 agar	0	1	15	1	0	32	1	0	0	50
M3 agar	0	0	0	0	0	0	14	0	0	14
OLIGO agar	57	0	0	11	0	68	5	1	45	187
GP agar	0	0	0	3	35	39	3	0	1	81
Mucin agar	101	3	18	79	65	5	1	0	29	301
Crenarchaeota agar	58	3	9	59	4	9	4	0	24	170
Total per sample	216	7	42	153	104	153	28	1	99	803
Total per depth	265			410			128			

B. *A. sventres*

Scraping isolates	*A. sventres* upper mesophotic			*A. sventres* shallow			Total per medium
	AS1	AS2	AS3	AS6	AS7	AS10	
MA1/10 agar	0	0	1	3	0	2	6
M3 agar	1	0	1	2	0	0	4
OLIGO agar	36	524	151	59	62	128	960
GP agar	0	0	94	0	4	0	98
Mucin agar	36	382	143	99	100	40	800
Crenarchaeota agar	29	728	149	44	63	147	1160
Total per sample	102	1634	539	207	229	317	3028
Total per depth	2275			753			

marine drugs

Table S4. Colonies picked from *X. muta* (panel A) and *A. sventres* (panel B) with their Colony Morphology Code (CMC), clade number or singleton (based on 16S rRNA gene sequence). In columns 4, 5, 6, and 7 taxonomic information of isolates based on a BLAST search with 16S rRNA genes is provided. Finally, the depth range of the origin sponge samples is provided in the last column. Table S4 is ordered based on depth category.

A. *X. muta*

NO	Isolate Code	CMC	Clade/singleton	Phylum/Class	Closest relative	Acc number	% identity	Depth
1	Mucin-18.XM4	22522	1	Alphaproteobacteria	*Erythrobacter flavus* strain VG1 chromosome, complete genome	CP022528.1	100%	Lower mesophotic
2	Mucin-6.XM5	10512	2	Actinobacteria	*Micrococcus luteus* strain trpE16 genome	CP007437.1	99%	Lower mesophotic
3	Mucin-23.XM5	10512	3	Gammaproteobacteria	*Alcanivorax* sp. MCCC 1A00973 16S ribosomal RNA gene, partial sequence	KU681505.1	99%	Lower mesophotic
4	Mucin-17.XM4	10531	7	Firmicutes	*Bacillus firmus* strain KP 16S ribosomal RNA gene, partial sequence	MH071301.1	99%	Lower mesophotic
5	Mucin-16.XM3	20321	10	Alphaproteobacteria	*Ruegeria* sp. CECT 5091 16S ribosomal RNA gene, partial sequence	MH023307.1	99%	Lower mesophotic
6	OLIGO-2.XM3	12313	15	Alphaproteobacteria	*Pseudovibrio denitrificans* strain Ab134 16S ribosomal RNA gene, partial sequence	KX990273.1	99%	Lower mesophotic
7	Mucin-19.XM5	20511	singleton	Alphaproteobacteria	*Erythrobacter* sp. DSW98 16S ribosomal RNA gene, partial sequence	DQ395681.1	99%	Lower mesophotic
	OLIGO-3.XM3	10513	singleton	Alphaproteobacteria	*Ruegeria* sp. CECT 5091 16S ribosomal RNA gene, partial sequence	MH023307.1	98%	Lower mesophotic

8	MA1/10-6.XM5	10523	singleton	Firmicutes	*Escherichia coli* strain E41-1 chromosome, complete genome	CP028483.1	99%	Lower mesophotic
9	MA1/10-10.XM5	10512	singleton	Firmicutes	*Bacillus licheniformis* strain HRBL-15TDI7 chromosome, complete genome	CP014781.1	99%	Lower mesophotic
10	MA1/10-11.XM5	10512	singleton	Actinobacteria	*Dermacoccus nishinomiyaensis* strain M25, complete genome	CP008889.1	99%	Lower mesophotic
11	OLIGO-6.XM3	10512	singleton	Alphaproteobacteria	*Pseudovibrio denitrificans* strain Ab134 16S ribosomal RNA gene, partial sequence	KX990273.1	100%	Lower mesophotic
12	Mucin-1.XM3	10512	singleton	Alphaproteobacteria	*Primorskyibacter insulae* strain SSK3-2 16S ribosomal RNA, partial sequence	NR_144598.1	99%	Lower mesophotic
13	OLIGO-23.XM3	12313	singleton	Gammaproteobacteria	*Alcanivorax* sp. MCCC 1A00973 16S ribosomal RNA gene, partial sequence	KU681505.1	97%	Lower mesophotic
14	OLIGO-4.XM3	20521	singleton	Gammaproteobacteria	*Shewanella* sp. STAB603 16S ribosomal RNA gene, partial sequence	JF825445.1	97%	Lower mesophotic
15	MA1/10-1.XM4	10511	singleton	Gammaproteobacteria	*Stenotrophomonas maltophilia* strain CCC10S 16S ribosomal RNA gene, partial sequence	MF375922.1	100%	Lower mesophotic
16	Mucin-3.XM3	20521	singleton	Gammaproteobacteria	*Halomonas salifodinae* 16S ribosomal RNA gene, partial sequence	JQ716249.1	97%	Lower mesophotic
17	Mucin-24.XM5	10512	3	Gammaproteobacteria	*Alcanivorax* sp. MCCC 1A00973 16S ribosomal RNA gene, partial sequence	KU681505.1	99%	Lower mesophotic
18	OLIGO-25.XM3	20511	11	Alphaproteobacteria	*Ruegeria* sp. ZGT108 16S ribosomal RNA gene, partial sequence	KP726355.1	99%	Lower mesophotic

19	OLIGO-5.XM3	10512	11	Alphaproteobacteria	*Ruegeria* sp. strain RKSG040 16S ribosomal RNA gene, partial sequence	MG799442.1	99%	Lower mesophotic
20	CR-1.XM5	10511	15	Alphaproteobacteria	*Pseudovibrio denitrificans* strain Ab134 16S ribosomal RNA gene, partial sequence	KX990273.1	99%	Lower mesophotic
21	Mucin-25.XM5	20321	15	Alphaproteobacteria	*Pseudovibrio* sp. strain RKSG039 16S ribosomal RNA gene, partial sequence	MG799441.1	100%	Lower mesophotic
22	OLIGO-24.XM3	12314	16	Alphaproteobacteria	*Ruegeria* sp. strain RKSG062 16S ribosomal RNA gene, partial sequence	MG799449.1	99%	Lower mesophotic
23	Mucin-4.XM5	22321	16	Alphaproteobacteria	*Ruegeria* sp. strain RKSG062 16S ribosomal RNA gene, partial sequence	MG799449.1	99%	Lower mesophotic
24	Mucin-5.XM5	22341	16	Alphaproteobacteria	*Ruegeria* sp. strain RKSG062 16S ribosomal RNA gene, partial sequence	MG799449.1	99%	Lower mesophotic
25	MA1/10-2.XM7	20511	singleton	Alphaproteobacteria	*Sphingobium yanoikuyae* strain S72 chromosome, complete genome	CP023741.1	100%	Upper mesophotic
26	Mucin-11.XM8	12341	singelton	Actinobacteria	*Microbulbifer cystodytense* 16S rRNA gene, isolate C1	AJ620879.1	98%	Upper mesophotic
27	Mucin-7.XM7	10512	singleton	Alphaproteobacteria	*Pseudovibrio* sp. strain RKSG039 16S ribosomal RNA gene, partial sequence	MG799441.1	100%	Upper mesophotic
28	Mucin-32.XM9	20531	singleton	Alphaproteobacteria	*Pseudovibrio* sp. strain RKSG039 16S ribosomal RNA gene, partial sequence	MG799441.1	100%	Upper mesophotic
29	Mucin-31.XM9	20521	singleton	Alphaproteobacteria	*Pseudovibrio* sp. strain RKSG039 16S ribosomal RNA gene, partial sequence	MG799441.1	100%	Upper mesophotic

30	Mucin-28.XM7	20531	singleton	Gammaproteobacteria	*Alcanivorax* sp. MCCC 1A00973 16S ribosomal RNA gene, partial sequence	KU681505.1	99%	Upper mesophotic
31	Mucin-8.XM7	20522	singleton	Alphaproteobacteria	Ruegeria sp. WJ45-6 16S ribosomal RNA gene, partial sequence	JX853816.1	96%	Upper mesophotic
32	Mucin-27.XM7	20511	singleton	Alphaproteobacteria	Pseudovibrio sp. strain RKSG039 16S ribosomal RNA gene, partial sequence	MG799441.1	98%	Upper mesophotic
33	GP-2.XM8	22311	2	Actinobacteria	*Micrococcus luteus* strain SGAir0127 chromosome, complete genome	CP025616.1	99%	Upper mesophotic
34	GP-1.XM9	10511	4	Firmicutes	*Staphylococcus pasteuri* strain JS7 chromosome, complete genome	CP017463.1	99%	Upper mesophotic
35	Mucin-9.XM7	22541	15	Alphaproteobacteria	*Pseudovibrio* sp. strain RKSG039 16S ribosomal RNA gene, partial sequence	MG799441.1	99%	Upper mesophotic
36	Mucin-26.XM7	20331	15	Alphaproteobacteria	*Pseudovibrio* sp. strain RKSG039 16S ribosomal RNA gene, partial sequence	MG799441.1	99%	Upper mesophotic
37	CR-3.XM7	10211	15	Alphaproteobacteria	*Pseudovibrio* sp. strain RKSG039 16S ribosomal RNA gene, partial sequence	MG799441.1	100%	Upper mesophotic
38	Mucin-29.XM7	10534	15	Alphaproteobacteria	*Pseudovibrio* sp. strain RKSG039 16S ribosomal RNA gene, partial sequence	MG799441.1	100%	Upper mesophotic
39	Mucin-10.XM8	24541	15	Alphaproteobacteria	*Pseudovibrio* sp. strain RKSG039 16S ribosomal RNA gene, partial sequence	MG799441.1	99%	Upper mesophotic
40	Mucin-12.XM8	10512	15	Alphaproteobacteria	*Pseudovibrio* sp. strain RKSG039 16S ribosomal RNA gene, partial sequence	MG799441.1	99%	Upper mesophotic

41	Mucin-30.XM8	20513	15	Alphaproteobacteria	*Pseudovibrio* sp. strain RKSG039 16S ribosomal RNA gene, partial sequence	MG799441.1	100%	Upper mesophotic
42	CR-4.XM9	20422	15	Alphaproteobacteria	*Pseudovibrio* sp. strain RKSG039 16S ribosomal RNA gene, partial sequence	MG799441.1	100%	Upper mesophotic
43	MA1/10-7.XM9	22321	singleton	Firmicutes	*Macrococcus canis* strain KM45013, complete genome	CP021059.1	100%	Upper mesophotic
45	OLIGO-9.XM12	10512	11	Alphaproteobacteria	*Ruegeria pomeroyi* strain LS80 16S ribosomal RNA gene, partial sequence	FJ937909.1	99%	shallow
46	Mucin-33.XM15	20513	singleton	Alphaproteobacteria	*Pseudovibrio denitrificans* strain SCSIO_43753 16S ribosomal RNA gene, partial sequence	MH283837.1	100%	shallow
47	Mucin-36.XM15	30521	singleton	Gammaproteobacteria	*Vibrio* sp. VibC-Oc-063 16S ribosomal RNA gene, partial sequence	KF577116.1	96%	shallow
48	Mucin-20.XM12	20521	16	Alphaproteobacteria	Ruegeria sp. strain RKSG062 16S ribosomal RNA gene, partial sequence	MG799449.1	99%	shallow
49	GP-3.XM12	10513	singleton	Actinobacteria	*Curtobacterium pusillum* strain Nc5MA-2 16S ribosomal RNA gene, partial sequence	KP296214.1	99%	shallow
50	M3-1.XM12	20512	singleton	Actinobacteria	*Micrococcus luteus* 16S ribosomal RNA gene, partial sequence	KY007582.1	98%	shallow
51	OLIGO-12.XM15	10512	singleton	Alphaproteobacteria	*Erythrobacter vulgaris* strain 102-Py5 16S ribosomal RNA gene, partial sequence	MG456894.1	99%	shallow
52	OLIGO-26.XM12	32311	singleton	Alphaproteobacteria	*Ruegeria* sp. strain CCM43 16S ribosomal RNA gene, partial sequence	MF499123.1	99%	shallow

53	OLIGO-10.XM14	10511	singleton	Alphaproteobacteria	*Pseudovibrio denitrificans* strain A-253 16S ribosomal RNA gene, partial sequence	KC751041.1	98%	shallow
54	Mucin-34.XM15	20521	singleton	Alphaproteobacteria	*Pseudovibrio* sp. strain Bu15_11 16S ribosomal RNA gene, partial sequence	KY671136.1	97%	shallow
55	OLIGO-20.XM15	10512	singleton	Alphaproteobacteria	*Boseongicola* sp. MA-7-27 16S ribosomal RNA gene, partial sequence	KX268607.1	98%	shallow
56	CR-10.XM15	10522	singleton	Gammaproteobacteria	*Vibrio campbellii* CAIM 519 = NBRC 15631 strain ATCC 25920, CAIM 519T chromosome 1, complete sequence	CP015863.1	99%	shallow
57	OLIGO-16.XM15	10521	singleton	Gammaproteobacteria	*Gammaproteobacterium* clone S1-5-32 16S ribosomal RNA gene, partial sequence	KF786583.1	97%	shallow
58	MA1/10-3.XM12	20521	singleton	Firmicutes	*Bacillus pumilus* strain 145 chromosome, complete genome	CP027116.1	100%	shallow
59	OLIGO-22.XM15	20511	singleton	Firmicutes	*Oceanobacillus oncorhynchi* strain QTYC25b 16S ribosomal RNA gene, partial sequence59	KM974661.2	100%	shallow
60	OLIGO-11.XM15	10512	1	Alphaproteobacteria	*Erythrobacter flavus* strain VG1, complete genome	CP022528.1	100%	shallow
61	OLIGO-18.XM15	10512	11	Alphaproteobacteria	Marine *Alphaproteobacterium* L4-30 16S ribosomal RNA gene, partial sequence	KU170446.1	100%	shallow
62	OLIGO-13.XM15	22331	13	Actinobacteria	*Microbulbifer* sp. A4B17 gene for 16S rRNA, partial sequence	AB243106.1	99%	shallow
63	OLIGO-17.XM15	20513	16	Alphaproteobacteria	Ruegeria sp. strain RKSG062 16S ribosomal RNA gene, partial sequence	MG799449.1	99%	shallow

64	OLIGO-14.XM15	20312	singleton	Actinobacteria	Microbulbifer agarilyticus strain GP101, complete genome	GQ118704.1	96%	shallow
65	OLIGO-27.XM15	33431	13	Actinobacteria	*Microbulbifer* sp. A4B17 gene for 16S rRNA, partial sequence	AB243106.1	99%	shallow
66	OLIGO-7.XM12	32321	13	Gammaproteobacteria	*Pelagiobacter variabilis* partial 16S rRNA gene, strain A4	FM180508.1	99%	shallow
67	Mucin-22.XM15	24531	14	Alphaproteobacteria	*Ruegeria* sp. strain RKSG062 16S ribosomal RNA gene, partial sequence	MG799449.1	99%	shallow
68	OLIGO-8.XM12	20512	15	Alphaproteobacteria	*Pseudovibrio* sp. strain RKSG039 16S ribosomal RNA gene, partial sequence	MG799441.1	100%	shallow
69	Mucin-14.XM15	10511	15	Alphaproteobacteria	*Pseudovibrio* sp. strain RKSG039 16S ribosomal RNA gene, partial sequence	MG799441.1	99%	shallow
70	Mucin-35.XM15	10512	15	Alphaproteobacteria	*Pseudovibrio* sp. strain RKSG039 16S ribosomal RNA gene, partial sequence	MG799441.1	100%	shallow
71	Mucin-15.XM15	22341	16	Alphaproteobacteria	*Ruegeria* sp. strain RKSG062 16S ribosomal RNA gene, partial sequence	MG799449.1	100%	shallow
72	OLIGO-19.XM15	12513	16	Alphaproteobacteria	*Ruegeria* sp. strain RKSG062 16S ribosomal RNA gene, partial sequence	MG799449.1	100%	shallow
73	OLIGO-21.XM15	24514	16	Alphaproteobacteria	*Ruegeria* sp. strain RKSG062 16S ribosomal RNA gene, partial sequence	MG799449.1	100%	shallow
74	Mucin-21.XM15	30521	16	Alphaproteobacteria	*Alphaproteobacterium* sp. clone 12E11 16S ribosomal RNA gene, partial sequence	KC668966.1	99%	shallow

75	OLIGO-15.XM15	22311	17	Actinobacteria	*Microbulbifer* sp. GB02-3 16S ribosomal RNA gene, partial sequence	GQ118704.1	99%	shallow
76	Mucin-13.XM15	20321	17	Actinobacteria	*Microbulbifer* sp. GB02-3 16S ribosomal RNA gene, partial sequence	GQ118704.1	99%	shallow

B. *A. sventres*

No	Isolate	CMC	Clades/singleton	Phyla/Class	Closest relative	Acc number	% identity	Depth
1	GP-3.AS3	10512	4	Firmicutes	*Staphylococcus pasteuri* strain ATCC 51129 16S ribosomal RNA gene, partial sequence	MG757632.1	100%	Upper mesophotic
2	GP-8.AS3	22311	5	Firmicutes	*Staphylococcus epidermidis* strain ISLP23 16S ribosomal RNA gene, partial sequence	MF125036.1	100%	Upper mesophotic
3	GP-6.AS3	10512	12	Firmicutes	*Staphylococcus haemolyticus* strain M2_0m_PrM_10 16S ribosomal RNA gene, partial sequence	KY742479.1	100%	Upper mesophotic
4	OLIGO-24.AS3	32331	13	Actinobacteria	*Microbulbifer* sp. A4B17 gene for 16S rRNA, partial sequence	AB243106.1	99%	Upper mesophotic
5	Mucin-12.AS2	32331	15	Alphaproteobacteria	*Pseudovibrio* sp. strain RKSG039 16S ribosomal RNA gene, partial sequence	MG799441.1	99%	Upper mesophotic
6	Mucin-47.AS2	10522	15	Alphaproteobacteria	*Pseudovibrio* sp. strain RKSG039 16S ribosomal RNA gene, partial sequence	MG799441.1	100%	Upper mesophotic
7	Mucin-46.AS1	10521	singleton	*Ruegeria*	*Ruegeria* sp. JZ11IS69	KC429844.1	99%	Upper mesophotic

8	Mucin-16.AS3	10531	singleton	Gammaproteobacteria	*Vibrio campbellii* strain BoB-90 chromosome 1, complete sequence	CP026315.1	98%	Upper mesophotic
9	OLIGO-15.AS1	30321	singleton	Actinobacteria	*Microbulbifer* sp. A4B17 gene for 16S rRNA, partial sequence	CP029064.1	99%	Upper mesophotic
10	OLIGO-14.AS1	22331	singleton	Actinobacteria	*Microbulbifer* sp. A4B17 gene for 16S rRNA, partial sequence	CP029064.1	96%	Upper mesophotic
11	Mucin-45.AS1	10521	11	Alphaproteobacteria	*Ruegeria pomeroyi* strain LS80 16S ribosomal RNA gene, partial sequence	FJ937909.1	100%	Upper mesophotic
12	OLIGO-16.AS1	10512	11	Alphaproteobacteria	*Ruegeria pomeroyi* strain LS80 16S ribosomal RNA gene, partial sequence	FJ937909.2	99%	Upper mesophotic
13	Mucin-9.AS1	10531	11	Proteobacteria	*Proteobacterium* clone L4-30 16S ribosomal RNA gene, partial sequence	KU170446.1	100%	Upper mesophotic
14	OLIGO-18.AS2	30321	13	Actinobacteria	*Microbulbifer* sp. A4B17 gene for 16S rRNA, partial sequence	CP029064.1	99%	Upper mesophotic
15	CR-5.AS1	20513	15	Alphaproteobacteria	*Pseudovibrio* sp. strain RKSG039 16S ribosomal RNA gene, partial sequence	MG799441.1	100%	Upper mesophotic
16	OLIGO-13.AS1	20511	15	Alphaproteobacteria	*Pseudovibrio* sp. strain RKSG039 16S ribosomal RNA gene, partial sequence	MG799441.1	99%	Upper mesophotic
17	Mucin-34.AS2	20331	15	Alphaproteobacteria	*Pseudovibrio* sp. strain RKSG039 16S ribosomal RNA gene, partial sequence	MG799441.1	99%	Upper mesophotic
18	Mucin-48.AS2	10521	15	Alphaproteobacteria	*Pseudovibrio* sp. strain RKSG039 16S ribosomal RNA gene, partial sequence	MG799441.1	99%	Upper mesophotic

19	OLIGO-17.AS2	20512	15	Alphaproteobacteria	*Pseudovibrio* sp. strain RKSG039 16S ribosomal RNA gene, partial sequence	MG799441.1	100%	Upper mesophotic
20	OLIGO-19.AS2	10511	15	Alphaproteobacteria	*Pseudovibrio* sp. strain RKSG039 16S ribosomal RNA gene, partial sequence	MG799441.1	99%	Upper mesophotic
21	OLIGO-20.AS2	10512	15	Alphaproteobacteria	*Pseudovibrio* sp. strain RKSG039 16S ribosomal RNA gene, partial sequence	MG799441.1	99%	Upper mesophotic
22	Mucin-14.AS3	22531	15	Alphaproteobacteria	*Pseudovibrio* sp. strain RKSG039 16S ribosomal RNA gene, partial sequence	MG799441.1	99%	Upper mesophotic
23	Mucin-35.AS3	24531	15	Alphaproteobacteria	*Pseudovibrio* sp. strain RKSG039 16S ribosomal RNA gene, partial sequence	MG799441.1	99%	Upper mesophotic
24	Mucin-49.AS3	10521	15	Alphaproteobacteria	*Pseudovibrio* sp. strain RKSG039 16S ribosomal RNA gene, partial sequence	MG799441.1	99%	Upper mesophotic
25	Mucin-50.AS3	10521	15	Alphaproteobacteria	*Pseudovibrio* sp. strain RKSG039 16S ribosomal RNA gene, partial sequence	MG799441.1	100%	Upper mesophotic
26	Mucin-51.AS3	14531	15	Alphaproteobacteria	*Pseudovibrio* sp. strain RKSG039 16S ribosomal RNA gene, partial sequence	MG799441.1	100%	Upper mesophotic
27	OLIGO-22.AS3	10511	15	Alphaproteobacteria	*Pseudovibrio* denitrificans strain Ab134 16S ribosomal RNA gene, partial sequence	KX990273.1	100%	Upper mesophotic
28	OLIGO-35.AS7	10513	14	Alphaproteobacteria	*Ruegeria* sp. WJ45-6 16S ribosomal RNA gene, partial sequence	JX853816.1	98%	shallow
29	OLIGO-37.AS10	22311	16	Alphaproteobacteria	*Ruegeria* sp. strain RKSG062 16S ribosomal RNA gene, partial sequence	MG799449.1	100%	shallow

30	Mucin-36.AS6	20321	singleton	Alphaproteobacteria	*Ochrobactrum daejeonense* strain KNDSS-Mac1 16S ribosomal RNA gene, partial sequence	KY471630.1	100%	shallow
31	OLIGO-28.AS6	10531	singleton	Actinobacteria	*Microbulbifer variabilis* strain Ni-2088 16S ribosomal RNA gene, partial sequence	NR_041021.1	99%	shallow
32	OLIGO-31.AS6	10512	singleton	Alphaproteobacteria	*Halomonas phoceae* strain CCUG 5096 16S ribosomal RNA gene, partial sequence	AY922995.1	96%	shallow
33	OLIGO-34.AS7	10511	singleton	Alphaproteobacteria	*Alphaproteobacterium* S948 16S ribosomal RNA gene, partial sequence	FJ215570.1	100%	shallow
34	Mucin-56.AS7	10322	singleton	Alphaproteobacteria	*Pseudovibrio* sp. FO-BEG1, complete genome	KY671135.1	98%	shallow
35	Mucin-41.AS10	10531	singleton	Alphaproteobacteria	*Pseudovibrio* sp. strain RKSG039 16S ribosomal RNA gene, partial sequence	MG799441.1	100%	shallow
36	GP-10.AS7	10512	singleton	Firmicutes	*Pediococcus acidilactici* strain NRCC1 16S ribosomal RNA gene, partial sequence	KU504251.1	100%	shallow
37	M3-1.AS6	10311	singleton	Gammaproteobacteria	*Enterobacter ludwigii* strain 30ft1b 16S ribosomal RNA gene, partial sequence	MG602668.1	99%	shallow
38	OLIGO-30.AS6	10511	11	Alphaproteobacteria	*Ruegeria* sp. ZGT108 16S ribosomal RNA gene, partial sequence	KP726355.1	99%	shallow
39	OLIGO-39.AS10	22311	11	Alphaproteobacteria	*Ruegeria* sp. strain RKSG208 16S ribosomal RNA gene, partial sequence	MG799470.1	100%	shallow
40	Mucin-42.AS10	10531	11	Alphaproteobacteria	*Ruegeria pomeroyi* strain LS80 16S ribosomal RNA gene, partial sequence	FJ937909.1	99%	shallow
41	M3-4.AS6	20312	12	Firmicutes	*Staphylococcus haemolyticus* JCM 2416 gene for 16S ribosomal RNA, partial sequence	LC383923.1	100%	shallow

42	OLIGO-21.AS2	22531	13	Actinobacteria	*Microbulbifer epialgicus* strain F-104 16S ribosomal RNA gene, partial sequence	NR_041493.1	99%	middle
43	OLIGO-27.AS6	32531	13	Alphaproteobacteria	*Alphaproteobacterium* clone A85 16S ribosomal RNA gene, partial sequence	GQ215680.1	98%	shallow
44	OLIGO-29.AS6	32321	13	Actinobacteria	*Microbulbifer* sp. A4B17 gene for 16S rRNA, partial sequence	AB243106.1	99%	shallow
45	OLIGO-38.AS10	20411	13	Actinobacteria	*Microbulbifer* sp. A4B17 gene for 16S rRNA, partial sequence	CP029064.1	99%	shallow
46	OLIGO-40.AS10	32321	13	Actinobacteria	*Microbulbifer* sp. A4B17 gene for 16S rRNA, partial sequence	CP029064.1	99%	shallow
47	OLIGO-36.AS10	22531	13	Actinobacteria	*Microbulbifer* sp. A4B17 gene for 16S rRNA, partial sequence	CP029064.1	99%	shallow
48	OLIGO-25.AS6	10512	15	Alphaproteobacteria	*Pseudovibrio* sp. strain RKSG039 16S ribosomal RNA gene, partial sequence	MG799441.1	99%	shallow
49	OLIGO-26.AS6	10511	15	Alphaproteobacteria	*Pseudovibrio* sp. strain RKSG039 16S ribosomal RNA gene, partial sequence	MG799441.1	100%	shallow
50	Mucin-55.AS6	22331	15	Alphaproteobacteria	*Pseudovibrio* sp. strain RKSG039 16S ribosomal RNA gene, partial sequence	MG799441.1	99%	shallow
51	Mucin-18.AS6	22331	15	Alphaproteobacteria	*Pseudovibrio* sp. strain RKSG039 16S ribosomal RNA gene, partial sequence	MG799441.1	99%	shallow
52	Mucin-19.AS6	20331	15	Alphaproteobacteria	*Pseudovibrio* sp. strain RKSG039 16S ribosomal RNA gene, partial sequence	MG799441.1	99%	shallow

53	Mucin-37.AS6	10512	15	Alphaproteobacteria	*Pseudovibrio* sp. strain RKSG039 16S ribosomal RNA gene, partial sequence	MG799441.1	100%	shallow
54	Mucin-38.AS6	10521	15	Alphaproteobacteria	*Pseudovibrio* sp. strain RKSG039 16S ribosomal RNA gene, partial sequence	MG799441.1	99%	shallow
55	Mucin-52.AS6	10521	15	Alphaproteobacteria	*Pseudovibrio* sp. strain RKSG039 16S ribosomal RNA gene, partial sequence	MG799441.1	100%	shallow
56	Mucin-53.AS6	10522	15	Alphaproteobacteria	*Pseudovibrio* sp. strain RKSG039 16S ribosomal RNA gene, partial sequence	MG799441.1	100%	shallow
57	Mucin-54.AS6	22311	15	Alphaproteobacteria	*Pseudovibrio* sp. strain RKSG039 16S ribosomal RNA gene, partial sequence	MG799441.1	99%	shallow
58	Mucin-21.AS7	10531	15	Alphaproteobacteria	*Pseudovibrio* sp. strain RKSG039 16S ribosomal RNA gene, partial sequence	MG799441.1	99%	shallow
59	Mucin-22.AS7	22331	15	Alphaproteobacteria	*Pseudovibrio* sp. strain RKSG039 16S ribosomal RNA gene, partial sequence	MG799441.1	100%	shallow
60	Mucin-23.AS7	10531	15	Alphaproteobacteria	*Pseudovibrio* sp. strain RKSG039 16S ribosomal RNA gene, partial sequence	MG799441.1	99%	shallow
61	Mucin-24.AS7	22331	15	Alphaproteobacteria	*Pseudovibrio* sp. strain RKSG039 16S ribosomal RNA gene, partial sequence	MG799441.1	99%	shallow
62	Mucin-26.AS7	22331	15	Alphaproteobacteria	*Pseudovibrio* sp. strain RKSG039 16S ribosomal RNA gene, partial sequence	MG799441.1	100%	shallow
63	Mucin-39.AS7	20531	15	Alphaproteobacteria	*Pseudovibrio* sp. strain RKSG039 16S ribosomal RNA gene, partial sequence	MG799441.1	99%	shallow

64	Mucin-40.AS7	10512	15	Alphaproteobacteria	*Pseudovibrio* sp. strain RKSG039 16S ribosomal RNA gene, partial sequence	MG799441.1	99%	shallow
65	OLIGO-33.AS7	10511	15	Alphaproteobacteria	*Pseudovibrio* sp. strain RKSG039 16S ribosomal RNA gene, partial sequence	MG799441.1	99%	shallow
66	Mucin-27.AS10	22531	15	Alphaproteobacteria	*Pseudovibrio* sp. strain RKSG039 16S ribosomal RNA gene, partial sequence	MG799441.1	99%	shallow
67	Mucin-28.AS10	10531	15	Alphaproteobacteria	*Pseudovibrio* sp. strain RKSG039 16S ribosomal RNA gene, partial sequence	MG799441.1	99%	shallow
68	Mucin-29.AS10	20321	15	Alphaproteobacteria	*Pseudovibrio* sp. strain RKSG039 16S ribosomal RNA gene, partial sequence	MG799441.1	99%	shallow

marine drugs

MDPI

Table S5. Sponge specimens that were used as inocula for cultivation.

No	Species	Sample Code	Actual Depth (m)	Depth category
1	*X. muta*	XM3	72	Lower mesophotic
2	*X. muta*	XM4	85	Lower mesophotic
3	*X. muta*	XM5	82	Lower mesophotic
4	*X. muta*	XM7	52	Upper mesophotic
5	*X. muta*	XM8	52	Upper mesophotic
6	*X. muta*	XM9	48	Upper mesophotic
7	*X. muta*	XM12	27	Shallow
8	*X. muta*	XM14	27	Shallow
9	*X. muta*	XM15	27	Shallow
10	*A. sventres*	AS1	54	Upper mesophotic
11	*A. sventres*	AS2	52	Upper mesophotic
12	*A. sventres*	AS3	52	Upper mesophotic
13	*A. sventres*	AS6	12	Shallow
14	*A. sventres*	AS7	12	Shallow
15	*A. sventres*	AS10	27	Shallow

marine drugs

Table S6. List of barcodes, linker, unitag sequences and accession numbers of sponge inoculums (co) and scraped bacterial communities. Panel A is information of *X. muta* and Panel B is information of *A. sventres*.

(A) List of barcodes, linker, unitag sequences and accession numbers of *X. muta* inoculums and scraped bacterial communities.

Sample code	Initial Barcode	Barcode sequence	Linker UniTag1	Linker UniTag2	Barcode + Linker UniTag1	Barcode + Linker UniTag2	Accession number
co.XM3	MiSeq-1	CTGGATAA	CT	TA	CTGGATAACT	CTGGATAATA	SRX3998982
co.XM4	MiSeq-2	ATAAGGTC	CT	TA	ATAAGGTCCT	ATAAGGTCTA	SRX3998983
co.XM5	MiSeq-3	AATAAGGA			AATAAGGA	AATAAGGA	SRX3998984
co.XM7	MiSeq-4	TACTTATC	T	A	TACTTATCT	TACTTATCA	SRX3998985
co.XM8	MiSeq-5	ATCTCAGT			ATCTCAGT	ATCTCAGT	SRX3998986
co.XM9	MiSeq-6	GTCAACGT	T	A	GTCAACGTT	GTCAACGTA	SRX3998987
co.XM12	MiSeq-7	ATGTTCCA			ATGTTCCA	ATGTTCCA	SRX3998888
co.XM14	MiSeq-8	CGTCTGAG	T	A	CGTCTGAGT	CGTCTGAGA	SRX3998887
co.XM15	MiSeq-9	CCAAGTCA			CCAAGTCA	CCAAGTCA	SRX3998890
GP.XM7	MiSeq-10	GGAGTATG	CT	TA	GGAGTATGCT	GGAGTATGTA	SRX3998889
GP.XM12	MiSeq-11	TGGTTGAC	ACT	CTA	TGGTTGACACT	TGGTTGACCTA	SRX3998884
GP.XM14	MiSeq-12	GCCTCATC	ACT	CTA	GCCTCATCACT	GCCTCATCCTA	SRX3998883
MA.XM3	MiSeq-15	CTTCATGG	T	A	CTTCATGGT	CTTCATGGA	SRX3998885
MA.XM5	MiSeq-49	TCAGGCGA	T	A	TCAGGCGAT	TCAGGCGAA	SRX3998892
MA.XM8	MiSeq-17	AGAGATAA			AGAGATAA	AGAGATAA	SRX3998891
MA.XM9	MiSeq-18	GGTCAGAT	ACT	CTA	GGTCAGATACT	GGTCAGATCTA	SRX3998899
MA.XM12	MiSeq-19	CCGTCTGC	ACT	CTA	CCGTCTGCACT	CCGTCTGCCTA	SRX3998900
MA.XM15	MiSeq-20	GGAGCGCA	T	A	GGAGCGCAT	GGAGCGCAA	SRX3998897
OLIGO.XM3	MiSeq-21	CGACCGCG			CGACCGCG	CGACCGCG	SRX3998898

OLIGO.XM7	MiSeq-22	CGTCCTCC	T	A	CGTCCTCCT	CGTCCTCCA	SRX3998895
OLIGO.XM8	MiSeq-50	AGTACTGA	T	A	AGTACTGAT	AGTACTGAA	SRX3998896
OLIGO.XM9	MiSeq-24	CGCGCCAG	T	A	CGCGCCAGT	CGCGCCAGA	SRX3998893
OLIGO.XM15	MiSeq-25	TAATACGT	ACT	CTA	TAATACGTACT	TAATACGTCTA	SRX3998894
CR.XM3	MiSeq-51	TCGTCGCC	ACT	CTA	TCGTCGCCACT	TCGTCGCCCTA	SRX3998901
CR.XM4	MiSeq-27	GGCCAGTA	T	A	GGCCAGTAT	GGCCAGTAA	SRX3998902
CR.XM5	MiSeq-28	CTGAGTTC	ACT	CTA	CTGAGTTCACT	CTGAGTTCCTA	SRX3998906
CR.XM7	MiSeq-29	TCTCTACC	ACT	CTA	TCTCTACCACT	TCTCTACCCTA	SRX3998905
CR.XM8	MiSeq-30	CCGAGGCG	CT	TA	CCGAGGCGCT	CCGAGGCGTA	SRX3998904
CR.XM9	MiSeq-31	GAAGCTCG	CT	TA	GAAGCTCGCT	GAAGCTCGTA	SRX3998903
CR.XM12	MiSeq-32	AAGGTAAG	CT	TA	AAGGTAAGCT	AAGGTAAGTA	SRX3998910
CR.XM15	MiSeq-33	ATGCGATT	CT	TA	ATGCGATTCT	ATGCGATTTA	SRX3998909
M3.XM12	MiSeq-34	AAGATCGT	ACT	CTA	AAGATCGTACT	AAGATCGTCTA	SRX3998908
M3.XM14	MiSeq-35	ATATAGGA			ATATAGGA	ATATAGGA	SRX3998907
Mucin.XM3	MiSeq-36	TATATTGA			TATATTGA	TATATTGA	SRX3998912
Mucin.XM4	MiSeq-37	TCCGACCT	ACT	CTA	TCCGACCTACT	TCCGACCTCTA	SRX3998911
Mucin.XM5	MiSeq-38	TCCAGCTC	T	A	TCCAGCTCT	TCCAGCTCA	SRX3998929
Mucin.XM7	MiSeq-39	GCTTGATG	CT	TA	GCTTGATGCT	GCTTGATGTA	SRX3998930
Mucin.XM8	MiSeq-40	GTAAGAAG	CT	TA	GTAAGAAGCT	GTAAGAAGTA	SRX3998931
Mucin.XM9	MiSeq-41	GAACGCTG	CT	TA	GAACGCTGCT	GAACGCTGTA	SRX3998932
Mucin.XM12	MiSeq-42	CTGACCGG	T	A	CTGACCGGT	CTGACCGGA	SRX3998925
Mucin.XM14	MiSeq-43	CATCAGTT	ACT	CTA	CATCAGTTACT	CATCAGTTCTA	SRX3998926
Mucin.XM15	MiSeq-44	ACCGGAAT	CT	TA	ACCGGAATCT	ACCGGAATTA	SRX3998927

(B) List of barcodes, linker, unitag sequences and accession numbers of *A.sventres* inoculums and scraped bacterial communities.

Sample code	Initial Barcode	Barcode sequence	Linker UniTag1	Linker UniTag2	Barcode + Linker UniTag1	Barcode + Linker UniTag2	Accession number
co.AS1	MiSeq-4	TACTTATC	T	A	TACTTATCT	TACTTATCA	SRX3998928
co.AS2	MiSeq-5	ATCTCAGT			ATCTCAGT	ATCTCAGT	SRX3998923
co.AS3	MiSeq-6	GTCAACGT	T	A	GTCAACGTT	GTCAACGTA	SRX3998924
co.AS6	MiSeq-7	ATGTTCCA			ATGTTCCA	ATGTTCCA	SRX3998934
co.AS7	MiSeq-8	CGTCTGAG	T	A	CGTCTGAGT	CGTCTGAGA	SRX3998933
co.AS10	MiSeq-9	CCAAGTCA			CCAAGTCA	CCAAGTCA	SRX3998936
OLIGO.AS1	MiSeq-49	TCAGGCGA	T	A	TCAGGCGAT	TCAGGCGAA	SRX3998935
OLIGO.AS2	MiSeq-17	AGAGATAA			AGAGATAA	AGAGATAA	SRX3998938
OLIGO.AS3	MiSeq-18	GGTCAGAT	ACT	CTA	GGTCAGATACT	GGTCAGATCTA	SRX3998937
OLIGO.AS6	MiSeq-19	CCGTCTGC	ACT	CTA	CCGTCTGCACT	CCGTCTGCCTA	SRX3998940
OLIGO.AS7	MiSeq-20	GGAGCGCA	T	A	GGAGCGCAT	GGAGCGCAA	SRX3998939
OLIGO.AS10	MiSeq-21	CGACCGCG			CGACCGCG	CGACCGCG	SRX3998942
CR.AS1	MiSeq-25	TAATACGT	ACT	CTA	TAATACGTACT	TAATACGTCTA	SRX3998941
CR.AS2	MiSeq-51	TCGTCGCC	ACT	CTA	TCGTCGCCACT	TCGTCGCCCTA	SRX3998955
CR.AS3	MiSeq-27	GGCCAGTA	T	A	GGCCAGTAT	GGCCAGTAA	SRX3998956
CR.AS6	MiSeq-28	CTGAGTTC	ACT	CTA	CTGAGTTCACT	CTGAGTTCCTA	SRX3998953
CR.AS7	MiSeq-29	TCTCTACC	ACT	CTA	TCTCTACCACT	TCTCTACCCTA	SRX3998954
CR.AS10	MiSeq-30	CCGAGGCG	CT	TA	CCGAGGCGCT	CCGAGGCGTA	SRX3998959
Mucin.AS1	MiSeq-34	AAGATCGT	ACT	CTA	AAGATCGTACT	AAGATCGTCTA	SRX3998960
Mucin.AS2	MiSeq-35	ATATAGGA			ATATAGGA	ATATAGGA	SRX3998957
Mucin.AS3	MiSeq-36	TATATTGA			TATATTGA	TATATTGA	SRX3998958
Mucin.AS6	MiSeq-37	TCCGACCT	ACT	CTA	TCCGACCTACT	TCCGACCTCTA	SRX3998961

Mucin.AS7	MiSeq-38	TCCAGCTC	T	A	TCCAGCTCT	TCCAGCTCA	SRX3998962
Mucin.AS10	MiSeq-39	GCTTGATG	CT	TA	GCTTGATGCT	GCTTGATGTA	SRX3998976
MA.AS3	MiSeq-42	CTGACCGG	T	A	CTGACCGGT	CTGACCGGA	SRX3998975
MA.AS6	MiSeq-43	CATCAGTT	ACT	CTA	CATCAGTTACT	CATCAGTTCTA	SRX3998974
GP.AS7	MiSeq-44	ACCGGAAT	CT	TA	ACCGGAATCT	ACCGGAATTA	SRX3998973
M3.AS2	MiSeq-45	TACCTACT	CT	TA	TACCTACTCT	TACCTACTTA	SRX3998977
